# Neoadjuvant Chemotherapy Versus Primary Debulking Surgery in FIGO Stage III and IV Epithelial Ovarian, Tubal or Peritoneal Cancer: A Systematic Review and Meta-Analysis

**DOI:** 10.3389/or.2022.10605

**Published:** 2022-09-27

**Authors:** Alexander A. Tzanis, Christos Iavazzo, Alexandros Hadjivasilis, Hara Tsouvali, George Α. Antoniou, Stavros A. Antoniou

**Affiliations:** ^1^ Department of Medicine, University of Thessaly, Larissa, Greece; ^2^ Department of Surgery, Filiates General Hospital, Filiates, Greece; ^3^ Metaxa Cancer Hospital, Piraeus, Greece; ^4^ First Department of Medical Oncology, Agios Savvas Cancer Hospital, Athens, Greece; ^5^ Cyprus International Institute for Environmental and Public Health, Cyprus University of Technology Limassol, Cyprus, Greece; ^6^ University Hospital of Ioannina, Ioannina, Greece; ^7^ Department of Vascular and Endovascular Surgery, Manchester University NHS Foundation Trust, Manchester, United Kingdom; ^8^ Division of Cardiovascular Sciences, School of Medical Sciences, University of Manchester, Manchester, United Kingdom; ^9^ Department of Surgery, Mediterranean Hospital of Cyprus, Limassol, Cyprus

**Keywords:** neoadjuvant chemotherapy, primary debulking surgery, epithelial ovarian cancer, tubal cancer, peritoneal cancer

## Abstract

**Objective:** To investigate whether neoadjuvant chemotherapy (NACT) confers superior outcomes compared to primary debulking surgery (PDS) in patients with stage III and IV epithelial ovarian, tubal or peritoneal cancer as well as in patients with high tumour load.

**Methods:** We searched the electronic databases PubMed, Cochrane Central Register of Controlled trials, and Scopus from inception to March 2021. We considered randomised controlled trials (RCTs) comparing NACT with PDS for women with epithelial ovarian cancer (EOC) stages III and IV. The primary outcomes were overall survival and progression-free survival. Secondary outcomes were optimal cytoreduction rates, peri-operative adverse events, and quality of life.

**Results:** Six RCTs with a total of 1901 participants were included. Meta-analysis demonstrated similar overall survival (HR = 0.96, 95% CI [0.86–1.07]) and progression-free survival (HR = 0.98, 95% CI [0.89–1.08]) between NACT and PDS. Subgroup analyses did not demonstrate higher survival for stage IV patients (HR = 0.88, 95% CI [0.71–1.09]) nor for patients with metastatic lesions >5 cm (HR = 0.86, 95% CI [0.69–1.08]) treated with NACT, albeit with some uncertainty due to imprecision. Similarly, no survival benefit was observed in the subgroup of patients with metastatic lesions >10 cm (HR = 0.94, 95% CI [0.78–1.12]). NACT was associated with significantly higher rates of complete cytoreduction (RR = 2.34, 95% CI [1.48–3.71]). Severe peri-operative adverse events were less frequent in the NACT arm (RR = 0.34, 95% CI [0.16–0.72].

**Conclusion:** Patients with stage III and IV epithelial ovarian cancer undergoing NACT or PDS have similar overall survival. NACT is likely associated with higher rates of complete cytoreduction and lower risk of severe adverse events and peri-operative death.

## Introduction

Ovarian cancer is the 2nd most common gynaecologic malignancy, yet the most lethal one (1). Due to the lack of effective screening, nearly 60% of all cases are diagnosed at metastatic, Federation of International Gynaecologists and Obstetricians (FIGO) stage IIIc and IV (2). As a result, the 5-year survival rate for women with metastatic epithelial ovarian cancer (EOC), tubal or peritoneal carcinoma is approximately 30% (1).

Since the introduction of platinum-based compounds in the early 1980's, the standard of treatment for women with EOC FIGO stage III and IV has been primary debulking surgery (PDS) followed by platinum-based adjuvant systemic chemotherapy. The principal objective of surgery is to achieve minimal residual disease, as evidence suggests that optimal cytoreduction (residual tumour <1 cm) is the most important prognostic factor of survival (3). However, patients with advanced-stage EOC often present with extensive metastatic disease, thus making primary debulking surgery an aggressive procedure associated with high peri-operative morbidity and mortality.

Given the aggressive nature of the current standard of treatment, there is growing interest in alternative therapeutic approaches that are associated with fewer adverse events, higher quality of life and non-inferior survival outcomes. According to evidence from randomised trials, neoadjuvant chemotherapy (NACT) followed by interval debulking surgery (IDS) could be such an alternative and appears to offer certain clinical advantages (4, 5). Currently, guidelines of the European Society of Gynaecologic Oncology (ESGO) recommend neoadjuvant chemotherapy only for patients whose performance status is not compatible with a radical surgery and for patients with unresectable disease (6). Previous meta-analyses did not investigate patient subgroups that could benefit from NACT.

In this study we aim to investigate whether neoadjuvant chemotherapy offers superior survival rates, less peri-operative morbidity and mortality and better quality of life compared to primary debulking surgery in patients with advanced epithelial ovarian cancer. Furthermore, we investigate the relative effectiveness and safety in the subgroup of women with advanced EOC, in which NACT could offer a survival benefit of greater magnitude.

## Methods

This systematic review is reported in accordance with the PRISMA 2020 guidelines (7).

### Protocol and Registration

The review protocol was developed in advance and was registered in PROSPERO (8). Details on the protocol of this systematic review can be accessed at https://www.crd.york.ac.uk/PROSPERO/display_record.php?ID=CRD42020155229.

### Literature Search Strategy

We searched in the electronic databases PubMed, Cochrane Central Register of Controlled trials and Scopus for articles published from inception to March 2021. The search terms were “ovarian neoplasms”, “ovarian cancer”, “antineoplastic agents”, “chemotherapy”, “neoadjuvant”, “preoperative”, “interval”, “debulking”, “adjuvant”, “postoperative”, “primary” and “surgery.” The search syntax applied on PubMed was constructed using the Cochrane highly sensitive search for randomised control trials (RCTs) (Supplementary Appendix). We also searched the grey literature for relevant studies (Open Grey). Finally, we tried to identify any related articles in the literature, either by scrutinising the references of relevant studies or by manually searching other sources, including Google Scholar. Only studies published in English were eligible for our review.

### Inclusion and Exclusion Criteria

We included studies following the PICOS model:

• Population: women over 18 years of age with newly diagnosed epithelial ovarian, tubal or peritoneal cancer, FIGO stages III and IV, confirmed by imaging and histological or cytological analysis.
• Intervention: neoadjuvant platinum-based chemotherapy followed by interval debulking surgery.
• Comparison: primary debulking surgery followed by platinum-based adjuvant chemotherapy.
• Outcomes: overall survival and progression-free survival were primary outcomes. Cytoreduction rates, peri-operative adverse events of grade 3 and 4, post-operative mortality, and quality of life (QoL) were secondary outcomes. The following sub-categories of cytoreduction rates were examined: complete cytoreduction (no macroscopic residual disease); residual disease between 0 and 1 cm; and optimal cytoreduction, which is cytoreduction to less than 1 cm residual disease and includes both previous sub-categories.
• Study design: we considered only RCTs.

Studies were excluded in the context of the following exclusion criteria: 1) patients diagnosed with recurrent disease (rather than newly diagnosed patients); 2) chemotherapy was not administered only systemically, but also intraperitoneally; 3) non-RCT studies, and 4) studies not published in English.

### Subgroup Analyses

We performed subgroup analyses for overall survival according to: 1) FIGO stage and 2) metastatic tumour size. More specifically, we investigated whether NACT offers superior survival in patients with stage III and stage IV disease, and in patients with metastatic tumour sizes <5 cm, between 5 and 10 cm, and >10 cm in diameter.

### Data Extraction

Data extraction and evaluation was conducted by two independent reviewers (AT and AH). Disagreements were discussed with the senior author (SA) in order to reach consensus. Data were collected from abstract, main manuscript, graphs, tables, supplementary material and/or trial protocol. The following data were collected: 1) trial characteristics including study design, year of publication, first author’s name, number of participating institutions, total number of participants enrolled, number of patients allocated in each arm, and duration of follow-up; 2) clinical information on patients’ age, FIGO staging, histologic subtypes, cancer antigen-125 (CA-125) values, chemotherapeutic agents used, route of administration and number of cycles, and surgical techniques used; and 3) outcome data including overall survival, progression-free survival, cytoreduction rates, peri-operative complications and time period within which they were assessed, and quality of life.

We contacted the primary authors of the included studies through email enquiring any complementary unpublished data. In case this was not possible, we utilized unpublished data reported in a Cochrane review investigating the efficacy and safety of NACT in epithelial ovarian cancer (9).

### Risk of Bias Assessment

We assessed the quality of the included studies per outcome using the revised version of the Cochrane risk-of-bias tool for randomised trials (RoB 2) (10). We assessed the studies included in our review for potential risk of bias in each outcome arising from: the randomisation process, deviations from the intended interventions, missing outcome data, measurement of the outcome, and selection of the reported result. Plots demonstrating the results of our assessment were created using the robvis tool (11).

### Statistical Synthesis

Time-to-event data meta-analyses were conducted for follow-up outcomes and the results were reported as summary hazard ratios (HRs) and associated 95% confidence intervals (CIs). Direct methods were applied to calculate individual study HR and standard error (SE) for specific outcome measures from reported HR with CIs or the logrank Observed minus Expected events (O-E) and the logrank variance (V) on the research arm of each trial (12,13). The inverse-variance method of meta-analysis was used. Dichotomous outcome data were summarized using the Mantel-Haenszel method by calculating the risk ratio (RR) and 95% CI. For continuous outcome data, the mean difference (MD) and 95% CI were calculated using the inverse-variance method. We created corresponding forest plots using the Review Manager software (RevMan 5.4) (14). We applied fixed effect analyses in the absence of substantial conceptual, statistical and visual heterogeneity, otherwise a random effects model was employed. We quantified the variability in effect estimates due to heterogeneity by calculating the I^2^.

Trial sequential analysis boundaries for the outcomes “grade III/IV adverse events” and “no residual disease” were constructed using the Land and DeMets method (15). For calculation of the information size, we considered *α* = 5% and *β* = 20%. The relative risk reduction was calculated based on the incidence of the outcomes in each group. Summary effects were presented as Z-values calculated upon two-sided significant testing. Both conventional testing boundaries and O'Brien-Fleming *α*-spending function boundaries were constructed, in addition to futility boundaries (16). The Trial Sequential Analysis software 0.9.5.10 Beta (Copenhagen Trial Unit, Copenhagen, Denmark) was used for trial sequential analyses.

### Assessment of the Quality of Evidence

The quality of evidence was assessed in line with the GRADE methodology (17). We presented the overall certainty of evidence for each outcome and subgroup analyses using the GRADEpro GDT software (18).

## Results

### Study Characteristics

Our search identified a total of 2438 articles. After initial screening and application of inclusion and exclusion criteria, we identified 9 reports of 6 eligible RCTs4, (19–25), that reported a total of 1901 patients. The PRISMA flow diagram can be accessed at the supplementary appendix (Supplementary Figure S1).

Fagotti 2020 and Fagotti 2016 are reports of the single-institution SCORPION trial conducted in Italy. In the 2016 report, the authors presented the first analysis of peri-operative adverse events and QoL. In the 2020 report, with an additional recruit of participants, the authors present the survival analysis and the peri-operative morbidity with the additional participants. Participants were enrolled from 2011 to 2016 with a median follow-up of 5 years. One hundred ninety-nine women were deemed eligible for randomisation and 171 were randomised after staging laparoscopy and histologic confirmation. During staging laparoscopy, tumour load and resectability were assessed using a predictive index (PI) (26). Women with histological confirmation of stage IIIc and IV EOC, tubal or peritoneal cancer had to be assigned with a PI score between 8 and 12 in order to be randomised. If staging laparoscopy was deemed unfeasible, due to large masses occupying the abdominal cavity or infiltrating the abdominal wall, or if mesenteric retraction was present regardless the PI score, patients were withdrawn from the study before randomisation. Eighty-seven women were assigned to NACT, receiving 3 or 4 cycles of platinum-based chemotherapy prior to IDS, which was then followed by administration of the remaining cycles (2 or 3) to reach a total number of 6 cycles. Eighty-four women underwent PDS followed by 6 cycles of platinum-based chemotherapy. Patients assigned to PDS arm did not undergo additional cytoreductive surgery, according to the protocol. For quantitative synthesis, we extracted QoL data from Fagotti 2016 and data regarding survival outcomes and peri-operative morbidity from Fagotti 2020.

Onda 2020 and Onda 2016 are reports of the JCOG0602 multi-centre trial conducted in Japan presenting distinct outcomes. Thirty-four participating institutions enrolled 301 patients with stage III and IV EOC, tubal or peritoneal cancer between 2006 and 2011. The follow-up period was 6 years. Diagnosis was made using clinical, radiological and cytological findings, without performing a diagnostic laparoscopy or laparotomy prior to randomisation. CA-125 levels had to be greater than 200 U/mL and carcinoembryonic antigen (CEA) <20 ng/ml in order to rule out malignancies originating from the gastrointestinal tract. One hundred fifty-two patients were assigned to NACT, receiving 4 cycles of platinum-based chemotherapy prior to IDS, which was followed by 4 further cycles of platinum-based chemotherapy. One hundred forty-nine patients were assigned to PDS, followed by 8 cycles of platinum-based chemotherapy. If optimal cytoreduction (residual tumour <1 cm) was not achieved during PDS, an additional debulking attempt was allowed according to the protocol. Primary outcome was overall survival (reported by Onda 2020), and secondary outcome endpoints included progression-free survival (reported by Onda 2020) and treatment invasiveness reported as peri-operative adverse events. No QoL data were reported.

Kehoe 2015 (CHORUS trial) was a multi-centre randomised trial conducted in the United Kingdom and New Zealand. Eighty-seven participating institutions enrolled a total of 552 women from 2004 to 2010, with a median follow-up period of 4.4 years. Patients with clinical and radiological evidence of stage III or IV EOC, tubal or peritoneal cancer and a CA-125 to CEA ratio >25 were randomised either to receive PDS followed by 6 cycles of platinum-based chemotherapy or 3 cycles of platinum-based chemotherapy followed by IDS and a final 3-cycle platinum-based chemotherapy regimen. A total of 274 women were assigned to the NACT arm, and 276 women were assigned to the PDS arm. If optimal cytoreduction (residual disease <1 cm) was not achieved during PDS, an additional debulking surgery could be attempted after 3 cycles of chemotherapy according to the protocol. The primary outcome measure was overall survival, and secondary outcomes were progression-free survival and QoL. Even though peri-operative morbidity was not an endpoint of this study, data on adverse events were reported and were included in our quantitative synthesis.

Vergote 2010 and Greimel 2013 are reports of the EORTC trial presenting survival and QoL data, respectively. This is the first international, multi-centre, randomised trial that investigated the role of NACT in patients with advanced stage EOC, tubal or peritoneal cancer. Fifty-nine participating institutions enrolled 718 women with stage IIIc and IV EOC, tubal or peritoneal cancer from 1998 to 2006. Due to some authorization irregularities, 48 women were excluded, thus 670 women were finally randomised. Patients were randomised after biopsy-confirmed stage IIIc or IV EOC. If biopsy was not possible, clinical characteristics plus a fine needle aspiration (FNA) suggestive of adenocarcinoma and a CA-125 to CEA ratio >25 was utilized. Subsequently, three hundred and thirty-four women were assigned to NACT, receiving 3 cycles of platinum-based systemic chemotherapy, followed by IDS, and then another 3 cycles of platinum-based chemotherapy. Three hundred thirty-six women received PDS, followed by 6 cycles of platinum-based systemic chemotherapy. If optimal cytoreduction (residual disease <1 cm) was not achieved during PDS, a secondary debulking effort was permitted, if stable disease or response to chemotherapy was documented. Primary outcome was overall survival, while progression-free survival, peri-operative morbidity and mortality, and QoL were secondary outcomes. Of those, QoL was reported by Greimel 2013, and the rest of the outcomes were reported by Vergote 2010.

Kumar 2009 and Chekman 2015 were available in abstract form only. The former reports on an ongoing phase III randomised trial conducted in India, and data included in the abstract were part of an interim analysis of 128 participants. The authors included patients with stage IIIc and IV EOC (pleural effusion only—according to the protocol published at clinicaltrials.gov). Chekman 2015 reports a phase III randomised trial conducted in Algeria that recruited only patients (90 women) with stage IIIc EOC. Since the full text report was not available for either of the studies, few outcome data could be extracted, and the studies could not be fully assessed for possible sources of bias.

### Risk of Bias

Five studies provided sufficient data for time-to-event analyses. The overall risk of bias was low in 4 out of the 5 studies. Kumar 2009 (abstract only) was judged to be at high overall risk of bias since very limited information was provided to permit judgement. Regarding peri-operative complications, 5 studies provided data suitable for quantitative synthesis. Three were judged to be low overall risk of bias, and the other 2 were deemed high risk of bias. Finally, 2 studies reported QoL data suitable for meta-analysis. The overall risk of bias was high in both studies. Detailed risk of bias assessments and corresponding plots can be accessed at the supplementary appendix (Supplementary Figures S2–S9).

### Outcome Measures

#### Overall Survival and Progression-Free Survival

**FIGURE 1:**
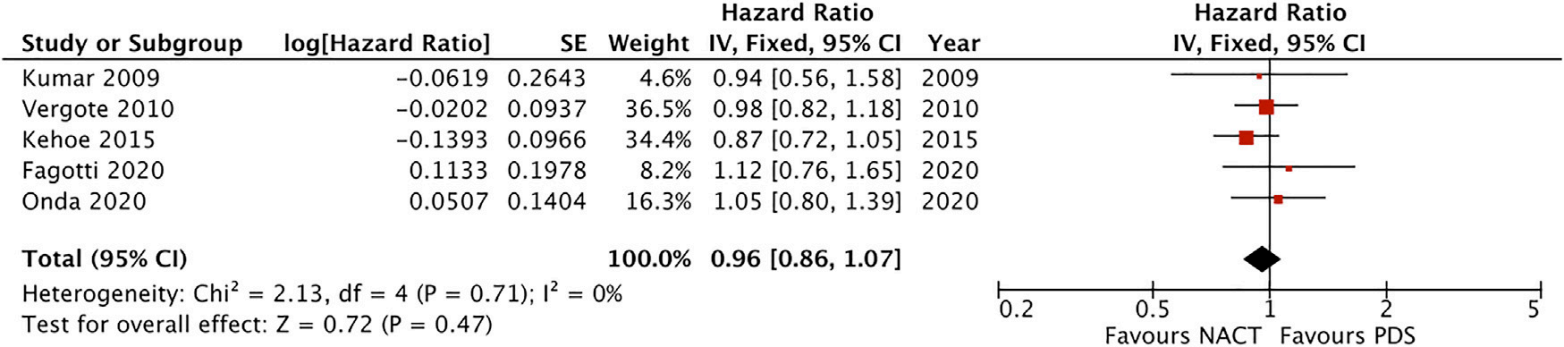
Forest plot for overall survival.

Five studies provided data on overall survival and progression-free survival (Kumar 2009, Vergote 2010, Kehoe 2015, Onda 2020 and Fagotti 2020). Meta-analysis of those studies, reporting a total of 1822 patients, suggested similar overall survival between NACT and PDS (HR = 0.96, 95% CI [0.86–1.07], I^2^ = 0%, favouring NACT) (Figure 1), and little difference in the risk of disease progression between the two interventions (HR = 0.98, 95% CI [0.89–1.08], I^2^ = 0%, favouring NACT). The certainty of evidence was high for both outcomes (Supplementary Table S1).

#### Cytoreduction Rates

Four studies provided data on residual disease after debulking surgery (PDS vs. IDS) and optimal cytoreduction rates achieved in each intervention arm (Vergote 2010, Kehoe 2015, Onda 2016 and Fagotti 2020). Statistical synthesis of those 4 studies was performed applying the intention-to-treat principle. A secondary as-treated analysis, including only patients that received surgery in both arms, is presented in the supplementary appendix (Supplementary Figure S11).

**FIGURE 2:**
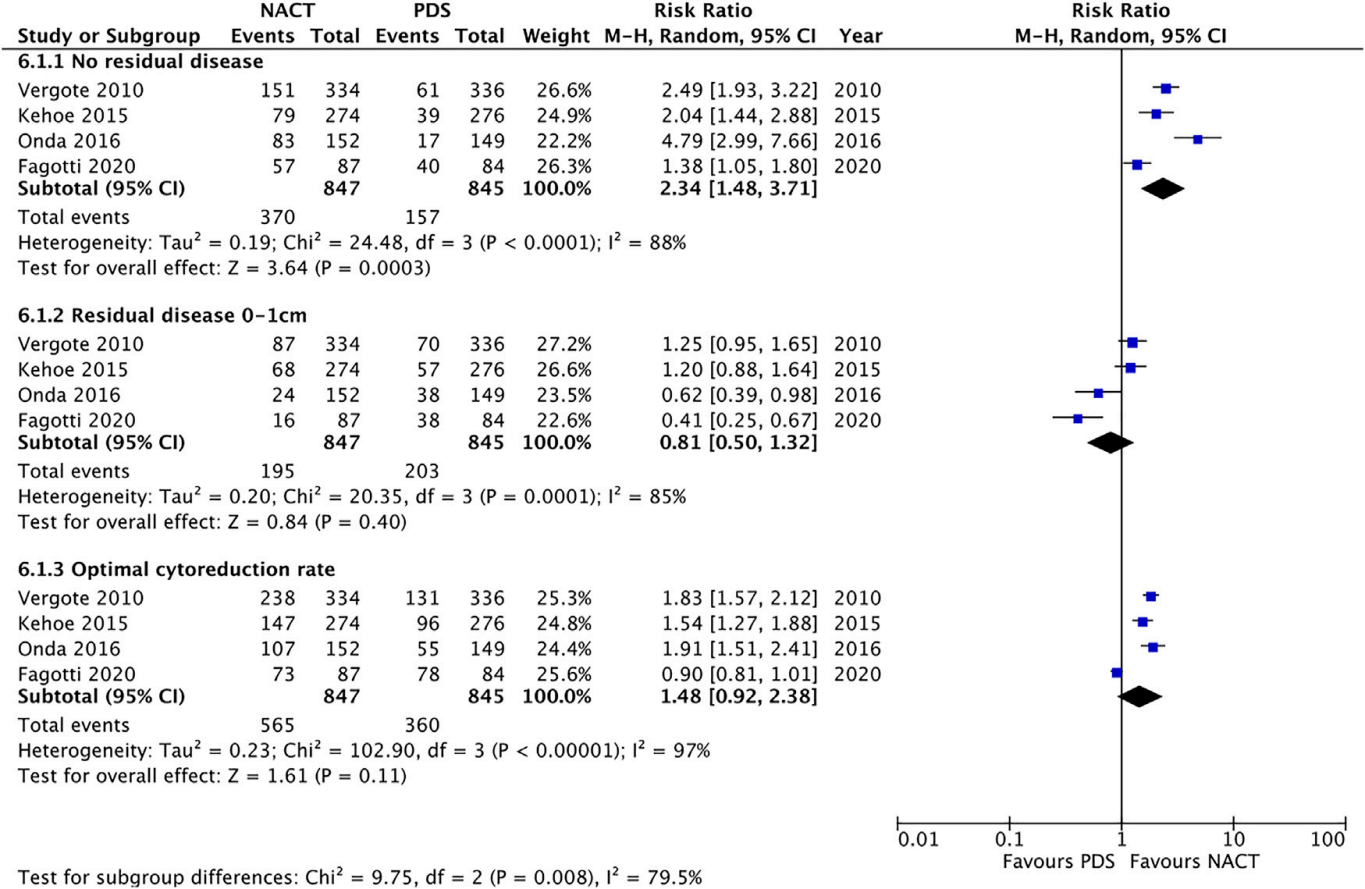
Forest plot for extent of residual disease after cytoreductive surgery.

Meta-analysis of 4 studies reporting outcomes for 1692 participants showed that NACT was associated with significantly higher rates of complete cytoreduction to no residual disease (RR = 2.34, 95% CI [1.48–3.71], I^2^ = 88%, moderate certainty of evidence), whereas no statistically important difference was found in the outcome optimal cytoreduction (RR = 1.48, 95% CI [0.92–2.38], I^2^ = 97%, moderate certainty of evidence). The risk of residual disease 0–1 cm was similar in the two groups (RR = 0.81, 95% CI from 0.5 to 1.32, I^2^ = 85%, low certainty of evidence) (Figure 2).

#### Peri-Operative Morbidity and Mortality

**FIGURE 3:**
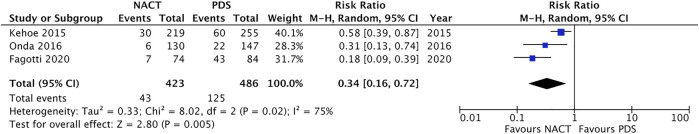
Forest plot for any grade 3 and 4 adverse event.

We investigated which of the two therapeutic approaches was associated with lower risk of severe (grade 3 and 4) peri-operative adverse events and post-operative mortality. Pooled analysis of 3 studies (Kehoe 2015, Fagotti 2020, Onda 2020) showed that NACT was associated with a statistically significant lower risk of grade 3 or 4 adverse events compared to PDS (RR = 0.34, 95% CI [0.16–0.72], I^2^ = 75%, moderate certainty of evidence) (Figure 3). When investigating specific grade 3 and 4 adverse events, NACT was associated with a lower risk of infection (RR = 0.28, 95% CI [0.15–0.52], I^2^ = 0%), venous thromboembolism (RR = 0.27, 95% CI [0.11–0.67], I^2^ = 18%), gastrointestinal fistula formation (RR = 0.26, 95% CI [0.06–1.02], I^2^ = 0%), and need for blood transfusion (RR = 0.85, 95% CI [0.75–0.96], I^2^ = 66%). No difference in the risk of haemorrhage was found between the two treatments (RR = 0.99, 95% CI [0.25–3.89], I^2^ = 84%). Finally, meta-analysis of 5 studies showed that NACT was associated with a significantly lower risk of post-operative mortality within 28 days (RR = 0.16, 95% CI [0.06–0.46], I^2^ = 0%) (Supplementary Figure S17).

#### Quality of Life

Three studies reported data on QoL data, but only 2 of them provided data suitable for meta-analysis (Greimel 2013 and Fagotti 2016). There was significant inconsistency in the reported results between the two studies, therefore the pooled analysis should be interpreted with caution. Regarding physical functioning, no difference was found between NACT and PDS at 6th cycle (MD = 3.39, 95% CI [−5.43 to 12.21], I^2^ = 99%), nor at 6-month follow-up (MD = −1.88, 95% CI [−4.62 to 0.86], I^2^ = 91%). Regarding global health, no clinically significant difference was observed between NACT and PDS at 6th cycle (MD = 0.98, 95% CI [0.49 to 1.48], I^2^ = 100%) and at 6-month follow-up (MD = −1.14, 95% CI [−1.67 to −0.61], I^2^ = 48%). NACT was associated with better QoL scores at 6 months follow-up in terms of role functioning (MD = 4.18, 95% CI [1.75 to 6.61], I^2^ = 84%) and emotional functioning (MD = 5.09, 95% CI [2.67 to 7.51], I^2^ = 86%). Further sub-categories of QoL scores were included in meta-analyses and are presented in the Supplementary Figures S18–S25.

### Subgroup Analyses

**FIGURE 4:**
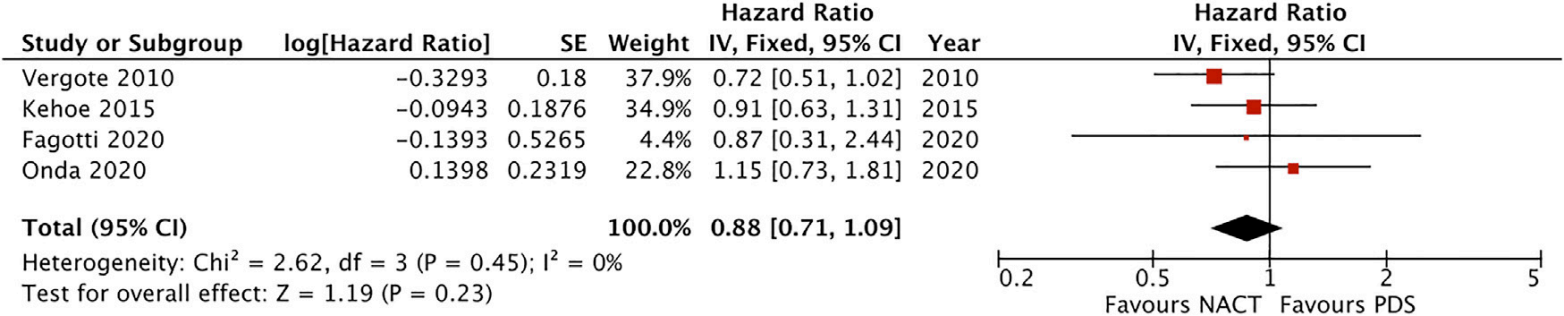
Forest plot for overall survival in the subgroup of patients with stage IV disease.

**FIGURE 5:**
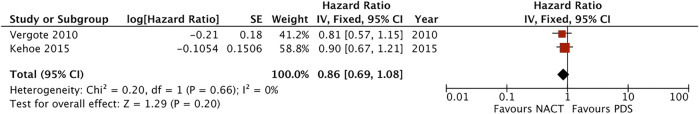
Forest plot for overall survival in the subgroup of patients with metastatic tumour size 5–10 cm.

**FIGURE 6:**
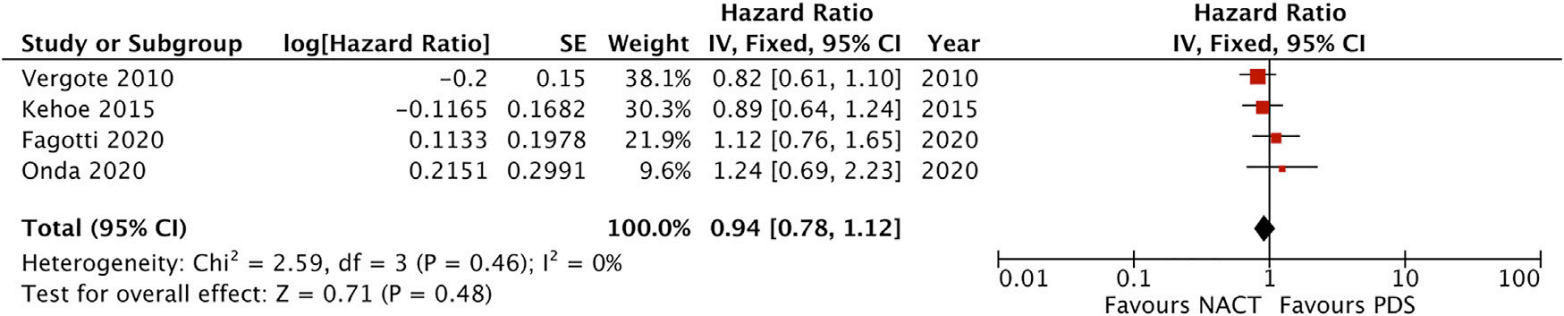
Forest plot for overall survival in the subgroup of patients with metastatic tumour size >10 cm.

In terms of survival by stage, pooled analysis of 4 studies showed no survival benefit with either approach in stage III patients (HR = 1.00, 95% CI [0.88–1.14], I^2^ = 2%, moderate certainty of evidence) (Supplementary Figure S26). Patients with stage IV EOC treated with NACT had better survival than those treated with PDS (HR = 0.88, 95% CI [0.71–1.09], I^2^ = 0%, moderate certainty of evidence), however it was not statistically significant (Figure 4). Subgroup analysis for metastatic tumour size did not suggest a statistically significant survival benefit for NACT in patients with metastatic lesions between 5 and 10 cm in diameter (HR = 0.86, 95% CI [0.69–1.08], I^2^ = 0%, moderate certainty of evidence) and in patients with metastatic lesions >10 cm (HR = 0.94, 95% CI [0.78–1.12], I^2^ = 0%, moderate certainty of evidence) (Figures 5, 6). No difference was found in patients with metastatic lesions ≤5 cm in diameter (HR = 1.09, 95% CI [0.72–1.64], I^2^ = 68%, low certainty of evidence) (Supplementary Figure S27).

### Trial Sequential Analyses

Trial sequential analyses for the outcomes “grade III/IV adverse events” and “no residual disease” suggested that evidence is likely definitive, as Z-curves have crossed *α*-spending boundaries (Supplementary Graphs 1–2). The information size was reached for the latter outcome, whereas another 137 patients are required for the information size to be reached regarding the outcome “grade III/IV adverse events.”

### Certainty of Evidence

Tables presenting a detailed certainty of evidence assessment of each outcome are provided within the Supplementary Appendix.

## Discussion

This meta-analysis demonstrated little difference in terms of overall survival and progression-free survival between NACT and PDS. When examining the overall survival by stage, we found that there was no difference between the two arms in patients with stage III disease. However, subgroup analyses of stage IV patients, patients with metastatic lesions >5 cm and patients with metastatic lesions >10 cm demonstrated a consistent trend in favour of NACT with no evidence of heterogeneity, albeit without statistical significance. Furthermore, no significant survival difference was observed by either approach in patients with metastatic tumour size <5 cm in diameter. These data suggest that the hypothesis that NACT might be more beneficial for patients with stage IV EOC or for patients with high tumour load should be further investigated.

NACT was also associated with higher complete cytoreduction rates (43.7% in the NACT arm vs. 18.6% in the PDS arm). The superiority of NACT was even more pronounced when data were pooled in an “as treated” analysis, including only patients that managed to receive surgery in both arms, suggesting higher efficiency as well as efficacy of NACT over PDS (no residual disease RR = 2.58, 95% CI [1.68–3.96], optimal cytoreduction rate RR = 1.64, 95% CI [0.93–2.88]). In terms of optimal cytoreduction rates, meta-analysis demonstrated significant heterogeneity among the included studies. Fagotti 2020 (SCORPION trial) was identified as the primary source of heterogeneity, reporting similar cytoreduction rates between the two arms. This could be attributed to two main reasons: 1) fewer stage IV women were recruited compared to the rest of the included trials (12% of the population) and 2) only patients with PI score between 8 and 12, suggestive of achieving optimal cytoreduction, were recruited 27.

Nonetheless, NACT seemed to offer superior rates of cytoreduction to no residual disease as reported by all 4 pooled trials, especially by EORTC, CHORUS and JCOG trials, where more patients with stage IV disease were included (24% in EORTC trial, 25% in CHORUS trial and 32% by JCOG trial). Complete cytoreduction was achieved in 12–19.4% of the patients in the PDS arm versus 39–57% in the NACT arm. The SCORPION trial reported significantly higher rates of complete cytoreduction in both arms compared to the rest of the included studies (47.6% in the PDS arm versus 77% in the NACT arm), likely due to recruiting participants who had higher chances of optimal debulking to be achieved. Higher cytoreduction rates were also achieved by the JCOG trial in the PDS arm, when accounting for the additional debulking surgeries that were introduced to women that did not achieve optimal cytoreduction during PDS, thus increasing the complete cytoreduction rate in the PDS arm to 31%. This introduction of an additional debulking surgery in the PDS arm in nearly 33% of the population (compared to 17% in the EORTC trial and 0% in the SCORPION trial) may be the cause of higher overall survival of patients in the PDS arm as reported by Onda et al. The reason behind the low cytoreduction rates reported by most included trials might be the selection of patients with bulky disease. Almost 70% of the population in the CHORUS trial had metastatic lesions >5 cm in diameter, while 40% of the EORTC trial and all patients in the SCORPION trial had metastatic lesions >10 cm in diameter. As a result, debulking rates and overall survival data in the general population should be interpreted with caution, making subgroup analyses according to stage of disease and metastatic tumour size even more important.

Even though NACT was associated with a significant improvement in complete cytoreduction rates, this was not translated into survival benefit in the general patient population. This could be attributed to two main reasons: 1) the low rates of complete cytoreduction, as discussed above, and 2) the increased chemo-resistance observed in women treated with NACT, especially those with high-grade serous ovarian carcinomas (28–30). Platinum-resistant or platinum-refractory disease is usually observed in women with high tumour load and serous subtype, as well as in women treated with multiple cycles of chemotherapy. In our study, this was reflected in the JCOG trial, where patients treated with NACT received 4 cycles of chemotherapy prior to IDS, in contrast with 3 cycles administered in the rest of the trials. Post-progression survival in the NACT arm of the JCOG trial was 6 months shorter than the PDS arm, suggesting that increased number of chemotherapy cycles induced higher rates of platinum resistance in the NACT arm.

As far as peri-operative morbidity is concerned, our results showed a lower risk of adverse events of grade 3 and 4 in patients treated with NACT. Ten percent of the patients randomised in the NACT arm experienced a grade 3 or 4 adverse event, whereas 25% of patients in the PDS arm faced some kind of severe adverse event. More specifically, NACT was associated with reduced risk of severe infections, venous thromboembolism, formation of gastrointestinal fistulas and need for blood transfusion—the latter being associated with worse prognosis—within 28 days post-surgery. Post-operative mortality was also significantly lower in the NACT arm (0.4% versus 3.3% in the PDS arm). Our results suggest that PDS is a more aggressive approach, associated with increased peri-operative morbidity and post-operative mortality within 28 days and seem to be consistent with the results reported by previous meta-analyses (9, 31, 32). Complete cytoreduction should remain the main goal of surgery, irrespective of the timing performed, as it is the single most important factor associated with increased survival in patients with advanced EOC33. However, maximal surgical effort during PDS in patients with high tumour load could impose increased morbidity or even death. In this context, NACT and IDS seem to offer a safer therapeutic approach associated with less adverse events.

Our study appears to have certain limitations, the most important of which is the heterogeneity between the included studies in certain outcomes, which however, on several occasions, could be conceptually explained. Different surgical techniques and outcomes were observed, not only between the trials, but also between institutions within the same trial. Moreover, recruitment of women in the included studies appeared to differ in certain parameters: some trials randomised patients after diagnostic laparoscopy or laparotomy (SCORPION trial) and other trials used a diagnostic surgery only in a small fraction of patients (EORTC and CHORUS trials), adding to the treatment invasiveness in both arms, while other studies (JCOG trial) did not use any invasive procedure prior to treatment initiation. Similarly, there is some degree of heterogeneity in the chemotherapeutic regimens used. Women participating in the JCOG trial received a target of 8 cycles of chemotherapy in both arms, while the rest of the trials administered a target-total of 6 cycles. Most trials aimed to recruit patients with bulky disease (SCORPION, EORTC and CHORUS trials), even some excluded patients with low tumour load (SCORPION trial), thus contributing to the observed heterogeneity. Finally, the use of additional first-line or maintenance treatment with vascular endothelial growth factor inhibitors (anti-VEGF), such as bevacizumab, or poly ADP-ribose polymerase inhibitors (PARP inhibitors), such as Olaparib, in patients with BRCA gene mutations, was either very limited (bevacizumab use in SCORPION trial) or absent.

In this context, the ongoing TRUST trial is recruiting patients with advanced EOC, irrespective of their tumour load. Furthermore, all participating institutions plan to meet certain criteria, such as complete cytoreduction rates during PDS ≥50%. The ongoing TRUST and SUNNY trials also utilize the approvement of anti-VEGF regimens, which could add in our existing knowledge (34, 35).

In conclusion, this meta-analysis demonstrated that NACT and PDS offer similar overall survival in patients with stage III and IV epithelial ovarian cancer. NACT seems to be associated with higher rates of complete cytoreduction and lower risk of severe adverse events and peri-operative death. Further research on the subgroups of patients with stage IV disease and those with high metastatic tumour load is warranted.

## Data Availability

The original contributions presented in the study are included in the article/Supplementary Material, further inquiries can be directed to the corresponding author.
