# Supplementary material for: Neoadjuvant Chemotherapy Versus Primary Debulking Surgery in FIGO Stage III and IV Epithelial Ovarian, Tubal or Peritoneal Cancer: A Systematic Review and Meta-Analysis
Source: Oncol Rev. 2022 Sep 27;16:10605. doi: 10.3389/or.2022.10605 (PMC9756845; doi:10.3389/or.2022.10605)
Supplement: Supplementary file 1 [file DataSheet2.pdf]

Author(s):
Question: Neoadjuvant chemotherapy compared to primary debulking surgery for women with stage III and IV epithelial ovarian cancer
Setting:
Bibliography:

Certainty assessment							№ of patients		Effect		Certainty	Importance
**№ of studies**	**Study design**	**Risk of bias**	**Inconsistency**	**Indirectness**	**Imprecision**	**Other considerations**	**neoadjuvant chemotherapy**	**primary debulking surgery**	**Relative (95% CI)**	**Absolute (95% CI)**		
**Overall survival (follow up: range 3 years to 6 years)**												
5	randomised trials	not serious	not serious	not serious	not serious	none	 -	 0.0%	**HR 0.96** (0.86 to 1.07) [Overall survival]	**-- per 1,000** (from -- to --)<br>**-- per 1,000** (from -- to --)	⨁⨁⨁⨁ HIGH	CRITICAL
**Progression-free survival (follow up: range 3 years to 6 years)**												
5	randomised trials	not serious	not serious	not serious	not serious	none	 -	 0.0%	**HR 0.98** (0.89 to 1.08) []	**-- per 1,000** (from -- to --)<br>**-- per 1,000** (from -- to --)	⨁⨁⨁⨁ HIGH	IMPORTANT
**Any grade 3 or 4 adverse event**												
3	randomised trials	not serious	not serious [a]	not serious	serious [b]	none	43/423 (10.2%)	125/486 (25.7%)	**RR 0.34** (0.16 to 0.72)	**170 fewer per 1,000** (from 216 fewer to 72 fewer)	⨁⨁⨁◯ MODERATE	CRITICAL
**Perioperative death within 28 days**												
5	randomised trials	not serious	not serious	not serious	serious [b]	none	3/786 (0.4%)	26/836 (3.1%)	**RR 0.16** (0.06 to 0.46)	**26 fewer per 1,000** (from 29 fewer to 17 fewer)	⨁⨁⨁◯ MODERATE	CRITICAL
**No residual disease**												
4	randomised trials	not serious	serious [c]	not serious	not serious	none	370/847 (43.7%)	157/845 (18.6%)	**RR 2.34** (1.48 to 3.71)	**249 more per 1,000** (from 89 more to 504 more)	⨁⨁⨁◯ MODERATE	CRITICAL
**Optimal cytoreduction rates**												
4	randomised trials	not serious	not serious [e]	not serious	serious [d]	none	565/847 (66.7%)	360/845 (42.6%)	**RR 1.48** (0.92 to 2.38)	**204 more per 1,000** (from 34 fewer to 588 more)	⨁⨁⨁◯ MODERATE	IMPORTANT
**Overall survival tumour size ≤5cm**												
3	randomised trials	not serious	serious [c]	not serious	serious [d]	none	 -	 0.0%	**HR 1.10** (0.87 to 1.38) [Overall survival tumour size ≤5cm]	**-- per 1,000** (from -- to --)<br>**-- per 1,000** (from -- to --)	⨁⨁◯◯ LOW	CRITICAL
**Overall survival tumour size 5-10cm**												
2	randomised trials	not serious	not serious	not serious	serious [d]	none	 -	 0.0%	**HR 0.86** (0.69 to 1.08) [Overall survival tumour size 5-10cm]	**-- per 1,000** (from -- to --)<br>**-- per 1,000** (from -- to --)	⨁⨁⨁◯ MODERATE	CRITICAL
**Overall survival tumour size >10cm**												
4	randomised trials	not serious	not serious	not serious	serious [d]	none	 -	 0.0%	**HR 0.94** (0.78 to 1.12) [Overall survival tumour size >10cm]	**-- per 1,000** (from -- to --)<br>**-- per 1,000** (from -- to --)	⨁⨁⨁◯ MODERATE	CRITICAL
**Overall survival stage III**												

4	randomised trials	not serious	not serious	not serious	serious [d]	none			**HR 1.00** (0.88 to 1.14) [Overall survival stage III]	**-- per 1,000** (from -- to --)	⨁⨁⨁◯ MODERATE	CRITICAL
							-	0.0%		**-- per 1,000** (from -- to --)		
**Overall survival stage IV**												
4	randomised trials	not serious	not serious	not serious	serious [d]	none			**HR 0.88** (0.71 to 1.09) [Overall survival stage IV]	**-- per 1,000** (from -- to --)	⨁⨁⨁◯ MODERATE	CRITICAL
							-	0.0%		**-- per 1,000** (from -- to --)		

CI: Confidence interval; **HR:** Hazard Ratio; **RR:** Risk ratio; **MD:** Mean difference

Explanations

a. Considerable heterogeneity was observed between the included studies, however confidence intervals were consistently lower than a maximum of RR 0.87.
b. Due to few events (<400).
c. Considerable statistical heterogeneity.
d. CI overlaps no effect or minimal important difference and suggests much uncertainty about the true effect.
e. Statistical heterogeneity exists between the SCORPION trial and the rest of the included studies. However, this can be attributed to the difference of the population recruited by the SCORPION trial, as they decided to exclude patients with unresectable disease prior to randomization.
