# Supplementary material for: Neoadjuvant Chemotherapy Versus Primary Debulking Surgery in FIGO Stage III and IV Epithelial Ovarian, Tubal or Peritoneal Cancer: A Systematic Review and Meta-Analysis
Source: Oncol Rev. 2022 Sep 27;16:10605. doi: 10.3389/or.2022.10605 (PMC9756845; doi:10.3389/or.2022.10605)
Supplement: Supplementary file 2 [file DataSheet1.docx]

Supplementary appendix

Table of Contents

[Cochrane highly sensitive search syntax 2](#_Toc71839510)

[Risk of bias detailed assessment 3](#_Toc71839511)

[Risk of bias plots 12](#_Toc71839512)

[Supplementary forest plots 15](#_Toc71839513)

[Trial sequential analyses 20](#_Toc71839514)

# Cochrane highly sensitive search syntax

(ovarian neoplasms [mh]) AND ((antineoplastic agents [mh]) OR chemotherapy) AND (neoadjuvant OR neo-adjuvant OR pre-operative OR preoperative OR interval) AND (postoperative OR post-operative OR adjuvant) AND (surgery [sh]) AND ((randomized controlled trial [pt]) OR (controlled clinical trial [pt]) OR (randomized [tiab]) OR (placebo [tiab]) OR (drug therapy [sh]) OR (randomly [tiab]) OR (trial [tiab]) OR (groups [tiab]) NOT (animals [mh] NOT humans [mh]))


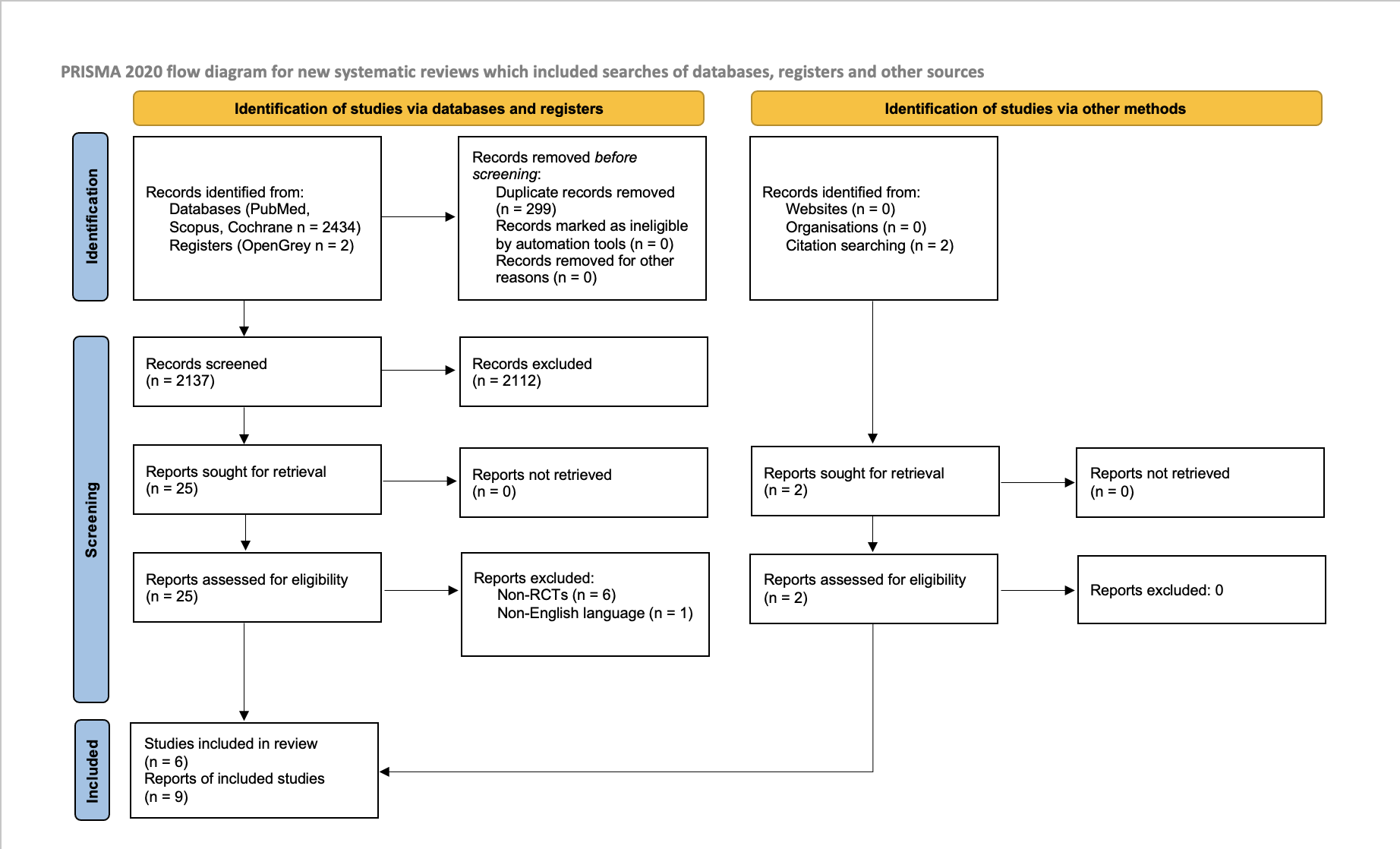


Figure 1. PRISMA flowchart of the literature search.

# Risk of bias detailed assessment

We did the assessment using the version 2 of the Cochrane risk of bias tool for randomized trials (RoB 2). Each domain of bias was judged as “low risk”, “high risk”, or “some concerns” according to the instructions of the revised tool.

9 studies were included in this meta-analysis and were all assessed for potential risks of bias. Of those studies, 3 couples are part of the same trial; (1) Fagotti et al. 2016 and Fagotti et al. 2018 are both part of the SCORPION trial, reporting perioperative adverse events and survival outcomes respectively, (2) Onda et al. 2016 and Onda et al. 2020 belong to the JCOG trial reporting perioperative complications and survival respectively, and (3) Vergote et al. 2010 and Greimel et al. 2013 are both part of the EORTC trial. The former reported perioperative adverse events and survival in both arms, while the latter delt with quality of life.

2 of the included studies (Chekman et al. 2015 and Kumar et al. 2009) were only available as abstracts. As a result, an in-depth risk of bias assessment was not possible.

1. **Fagotti et al. 2016 (SCORPION trial)**
   1. Domain 1. The risk of bias arising from the randomization process was judged to be low. The random assignment was centralised using a block-randomization computer-generated list. In addition, minor baseline differences were detected between the two intervention groups that could be attributed to chance.
   2. Domain 2. The risk of bias in regard to the effect of assignment to intervention was deemed low as a result of the intention-to-treat analysis that was conducted by the authors.

The risk of bias concerning the effect of adhering to intervention was also deemed to be low according to the algorithm.

- 1. Domain 3. The risk of bias because of missing outcome data was low for perioperative morbidity, but high for quality of life. Availability of data from at least 95% of participants was not present for quality-of-life analysis. Completion rates at different time points varied from 90.9% to 86.3%. Patients were excluded from quality-of-life analysis if they experienced progression of disease, which can be considered as a strong factor of deterioration of quality-of-life scores (missingness in the outcome depended on its true value).
  2. Domain 4. The risk of bias in measurement of the outcome was deemed low for perioperative morbidity and high risk for quality of life. No information was provided whether the outcome assessors were blinded to intervention received by participants. Even though this may not be important for assessment of perioperative morbidity (low risk of bias), it could raise serious concerns regarding the quality-of-life outcome measure, which is a participant-reported outcome.
  3. Domain 5. The risk of bias in selection of the reported result was low since there were no changes in the analysis plan.
  4. Overall risk of bias (assignment to intervention). The overall risk of bias was low for perioperative morbidity and high for quality-of-life (due to high risk of bias in domain 3 and 4).
  5. Overall risk of bias (adherence to intervention). The overall risk of bias was labelled as “some concerns” for perioperative morbidity and “high risk” for quality-of-life. Even though the tool’s algorithm suggests that there is a low risk of bias for the former, we judged that the absence of a per-protocol analysis could raise some concerns regarding the adherence to intervention.

1. **Fagotti et al. 2020 (SCORPION trial)**
   1. Domain 1. The risk of bias arising from the randomization process was judged to be low. The random assignment was centralised using a block-randomization computer-generated list. In addition, minor baseline differences were detected between the two intervention groups that could be attributed to chance.
   2. Domain 2. The risk of bias in regard to the effect of assignment to intervention was deemed low as a result of the intention-to-treat analysis that was conducted by the authors.

The risk of bias concerning the effect of adhering to the intervention was also deemed to be low. The authors of the study performed a per-protocol analysis in order to assess the treatment compliance.

- 1. Domain 3. The risk of bias because of missing outcome data was low for this study. Data were available for all randomized participants.
  2. Domain 4. The risk of bias in measurement of the outcome was deemed low across all outcome measures. Even though the authors of the study did not report whether the outcome assessors were blinded to assigned treatment, this could not influence the time-to-event outcomes or perioperative morbidity.
  3. Domain 5. The risk of bias in selection of the reported result was low since there were no changes in the analysis plan.
  4. Overall risk of bias (assignment to intervention). The overall risk of bias for this study was low.
  5. Overall risk of bias (adherence to intervention). The overall risk of bias for this study was low.

1. **Vergote et al. 2010 (EORTC trial)**
   1. Domain 1.  The risk of bias arising from the randomization process was judged to be low. Randomization was done centrally with the use of a minimization technique. In addition, no baseline differences between the characteristics of the intervention groups were detected.
   2. Domain 2. The risk of bias in regard to the effect of assignment to intervention was deemed low as a result of the intention-to-treat analysis that was conducted by the authors.

The risk of bias concerning the effect of adhering to the intervention was also deemed to be low. The authors of the study performed a secondary analysis based on the treatment actually received (per-protocol analysis) in order to assess the treatment compliance.

- 1. Domain 3. The risk of bias because of missing outcome data was low across all outcome measures (perioperative and postoperative morbidity and mortality, quality of life, and survival). Data were available for all randomized participants.
  2. Domain 4. The risk of bias in measurement of the outcome was deemed low. An independent data and safety monitoring committee was utilized for the purposes of monitoring the recruitment rate, the potential toxicity of the treatments, and the optimal percentage of debulking.
  3. Domain 5. The risk of bias in selection of the reported result was low. The study was conducted in accordance with the protocol as amended.
  4. Overall risk of bias (assignment to intervention). The overall risk of bias for this study was low.
  5. Overall risk of bias (adherence to intervention). The overall risk of bias for this study was low.

1. **Greimel et al. 2013 (EORTC trial)**

This study reported quality of life outcomes in selected population that participated in the EORTC trial. 404 patients were selected for quality-of-life analysis out of the 670 participants of the EORTC trial.

- 1. Domain 1. The risk of bias arising from the randomization process was judged to be low as indicated by Vergote et al. 2010.
  2. Domain 2. The risk of bias in regard to the effect of assignment to intervention as well as the risk of bias concerning the effect of adhering to the intervention for the EORTC trial were deemed to be low.
  3. Domain 3. The risk of bias because of missing outcome data was deemed high. The selection of the 404 patients for quality-of-life analysis originally gave rise to some concerns about the selection criteria. It could be possible that participants with better compliance or with overall greater satisfaction with the treatment received might have been selected.

These concerns were validated by the authors of the study. They compared the selected group of patients to the overall population of 670 participants of the EORTC trial to investigate potential systematic differences that could confirm bias. Statistically significant differences were found in the overall debulking rates between the two groups and institutions with poor debulking rates were excluded (39.9% optimal debulking in selected institutions versus 19.9% in excluded institutions [p=0.0011]). As a result, patients from selected institutions appeared to have better overall survival (32.3 months versus 23.29 months in excluded institutions [p=0.0006]) as well as better progression free survival (12.35 months versus 9.92 months in excluded institutions [p=0.0002]). Statistically significant differences were also observed in additional clinical characteristics like the tumour load, the use of fine needle aspiration as a diagnostic approach etc.

- 1. Domain 4. The risk of bias in measurement of the outcome was deemed high since quality-of-life is an outcome reported by participants of an open-label study.
  2. Domain 5. The risk of bias in selection of the reported result was labelled as “some concerns”. Due to the fact that compliance was too restrictive, changes were made to the protocol defined analysis plan, which resulted in the exclusion of many participants.
  3. Overall risk of bias (assignment to intervention). The overall risk of bias for this study was high due to high risk of bias in domains 3 and 4.
  4. Overall risk of bias (adherence to intervention). The overall risk of bias for this study was high due to high risk of bias in domains 3 and 4.

1. **Kehoe et al. 2015**
   1. Domain 1. The risk of bias arising from the randomization process was judged to be low. Randomization was done centrally with the use of a minimization technique. In addition, no baseline differences between the characteristics of the intervention groups were detected.
   2. Domain 2. The risk of bias in regard to the effect of assignment to intervention was deemed low as a result of the intention-to-treat analysis that was conducted by the authors.

The risk of bias concerning the effect of adhering to the intervention was also deemed to be low. The authors of the study performed a secondary analysis based on the treatment actually received (per-protocol analysis) in order to assess the treatment compliance.

- 1. Domain 3. The risk of bias because of missing outcome data was low across all outcome measures (overall survival, progression-free survival, perioperative complications, and quality of life). Data were available for all randomized participants.
  2. Domain 4. The risk of bias in measurement of the outcome was deemed low for survival outcomes and perioperative morbidity. Even though the authors of the study did not report whether the outcome assessors were blinded to assigned treatment, this could not influence the assessment of time-to-event data and perioperative morbidity. Regarding quality-of-life data, there are some concerns, since it is a participant-reported outcome. However, this study did not provide quality-of-life data that could be used in quantitative synthesis.
  3. Domain 5. The risk of bias in selection of the reported result was low since there were no changes in the analysis plan.
  4. Overall risk of bias (assignment to intervention). The overall risk of bias for this study was low.
  5. Overall risk of bias (adherence to intervention). The overall risk of bias for this study was low.

1. **Onda et al. 2016 (JCOG0602 trial)**
   1. Domain 1. The risk of bias arising from the randomization process was judged to be low. Randomization was done using a minimization technique. In addition, no baseline differences between the characteristics of the intervention groups were detected.
   2. Domain 2. The risk of bias in regard to the effect of assignment to intervention was deemed low.

The risk of bias concerning the effect of adhering to the intervention was high. There was an imbalance of some off-protocol surgeries that were performed in a small number of patients (1 in group A versus 13 in group B). In addition, the authors did not conduct a per-protocol analysis in order to assess the compliance in this study.

- 1. Domain 3. The risk of bias because of missing outcome data was low for this study. Data were available for all randomized participants.
  2. Domain 4. The risk of bias in measurement of the outcome was deemed low. Despite the fact that the outcome assessors were not masked to the intervention assigned to each group, the report of perioperative outcomes (amount of blood loss, infection rates, bowel obstruction etc) could not be affected by knowledge of the intervention received.
  3. Domain 5. The risk of bias in selection of the reported result was low since there were no changes in the analysis plan.
  4. Overall risk of bias (assignment to intervention). The overall risk of bias for this study was low.
  5. Overall risk of bias (adherence to intervention). The overall risk of bias for this study was high due to high risk of bias judgement in domain 2 (effect of adhering to intervention).

1. **Onda et al. 2020 (JCOG0602 trial)**
   1. Domain 1. The risk of bias arising from the randomization process was judged to be low. Randomization was done using a minimization technique. In addition, no baseline differences between the characteristics of the intervention groups were detected.
   2. Domain 2. The risk of bias in regard to the effect of assignment to intervention was deemed low.

The risk of bias concerning the effect of adhering to the intervention was high. There was an imbalance of some off-protocol surgeries that were performed in a small number of patients (1 in group A versus 13 in group B). In addition, the authors did not conduct a per-protocol analysis in order to assess the compliance in this study.

- 1. Domain 3. The risk of bias because of missing outcome data was low for this study. Data were available for all randomized participants.
  2. Domain 4. The risk of bias in measurement of the outcome was deemed low. Despite the fact that the outcome assessors were not masked to the intervention assigned to each group, the assessment of survival outcomes could not be affected by knowledge of the intervention received.
  3. Domain 5. The risk of bias in selection of the reported result was low since there were no changes in the analysis plan.
  4. Overall risk of bias (assignment to intervention). The overall risk of bias for this study was low.
  5. Overall risk of bias (adherence to intervention). The overall risk of bias for this study was high due to high risk of bias judgement in domain 2 (effect of adhering to intervention).

1. **Chekman et al. 2015**
   1. Domain 1. The risk of bias arising from the randomization process was judged as raising “some concerns”. The authors of the study mention that patients were randomized in two different arms, without providing information about the method of randomization.
   2. Domain 2. The risk of bias was deemed to be high both in regard to the effect of assignment to intervention and in regard to the effect of adhering to intervention. There was no information about the type(s) of analyses that were used for each outcome measure.
   3. Domain 3. The risk of bias because of missing outcome data was high. 9 patients were excluded during the randomization process for unknown reasons.
   4. Domain 4. The risk of bias in measurement of the outcome was labelled as “some concerns”. There is no information whether the outcome assessors were blinded to the assigned intervention. However, observation of perioperative morbidity could remain uninfluenced by knowledge of intervention received by each patient.
   5. Domain 5. The risk of bias in selection of the reported result was judged to raise “some concerns”. There is no sufficient information about any pre-specified analysis plan and whether any alterations were made on it.
   6. Overall risk of bias: The overall risk of bias for this study was high.
2. **Kumar et al. 2009**
   1. Domain 1. The risk of bias arising from the randomization process was judged as raising “some concerns”. The authors of the study mention that patients were randomized in two different arms, without providing information about the method of randomization.
   2. Domain 2. The risk of bias was deemed to be high both in regard to the effect of assignment to intervention and in regard to the effect of adhering to intervention. There was no information about the type(s) of analyses that were used for each outcome measure.
   3. Domain 3. The risk of bias because of missing outcome data was high. Information about any missing data is insufficient.
   4. Domain 4. The risk of bias in measurement of the outcome was labelled as “some concerns”. There is no information whether the outcome assessors were blinded to the assigned intervention. However, time-to-event data cannot be influenced by knowledge of the intervention received by each patient.
   5. Domain 5. The risk of bias in selection of the reported result was judged to raise “some concerns”. There is no sufficient information about any pre-specified analysis plan and whether any alterations were made on it.
   6. Overall risk of bias: The overall risk of bias for this study was high.

# Risk of bias plots

#
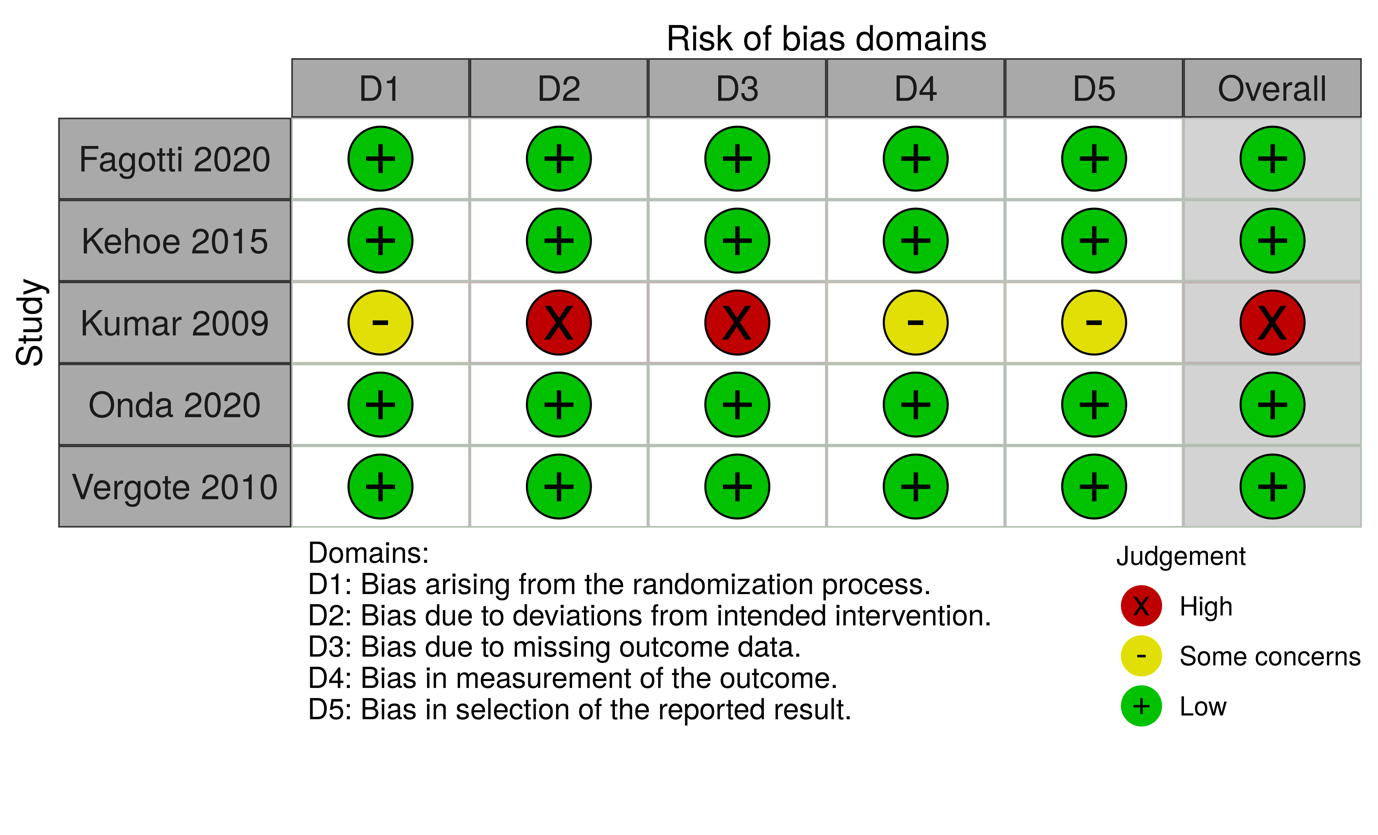


Figure 2. Time-to-event data, traffic-light plot.


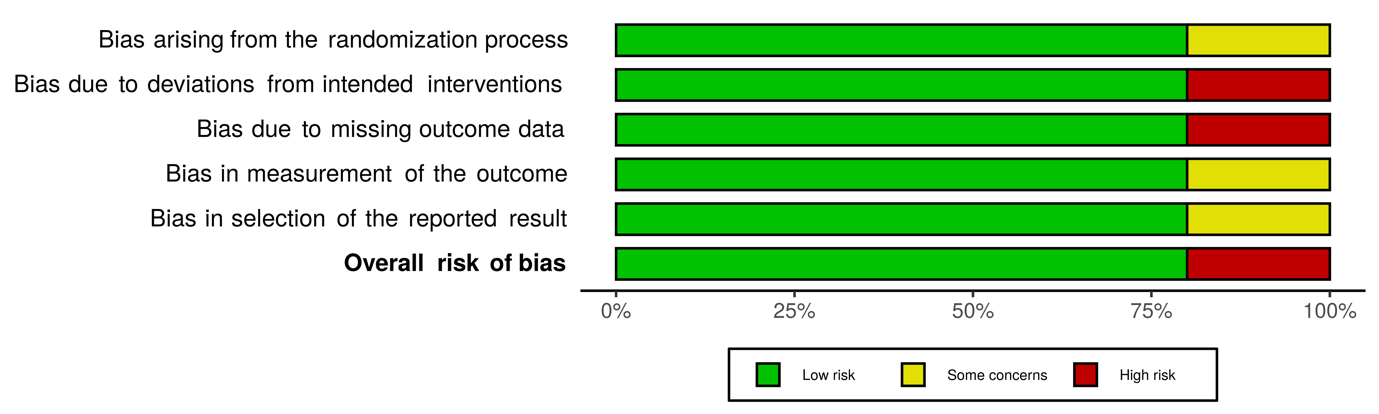


Figure 3. Time-to-event data, summary plot.


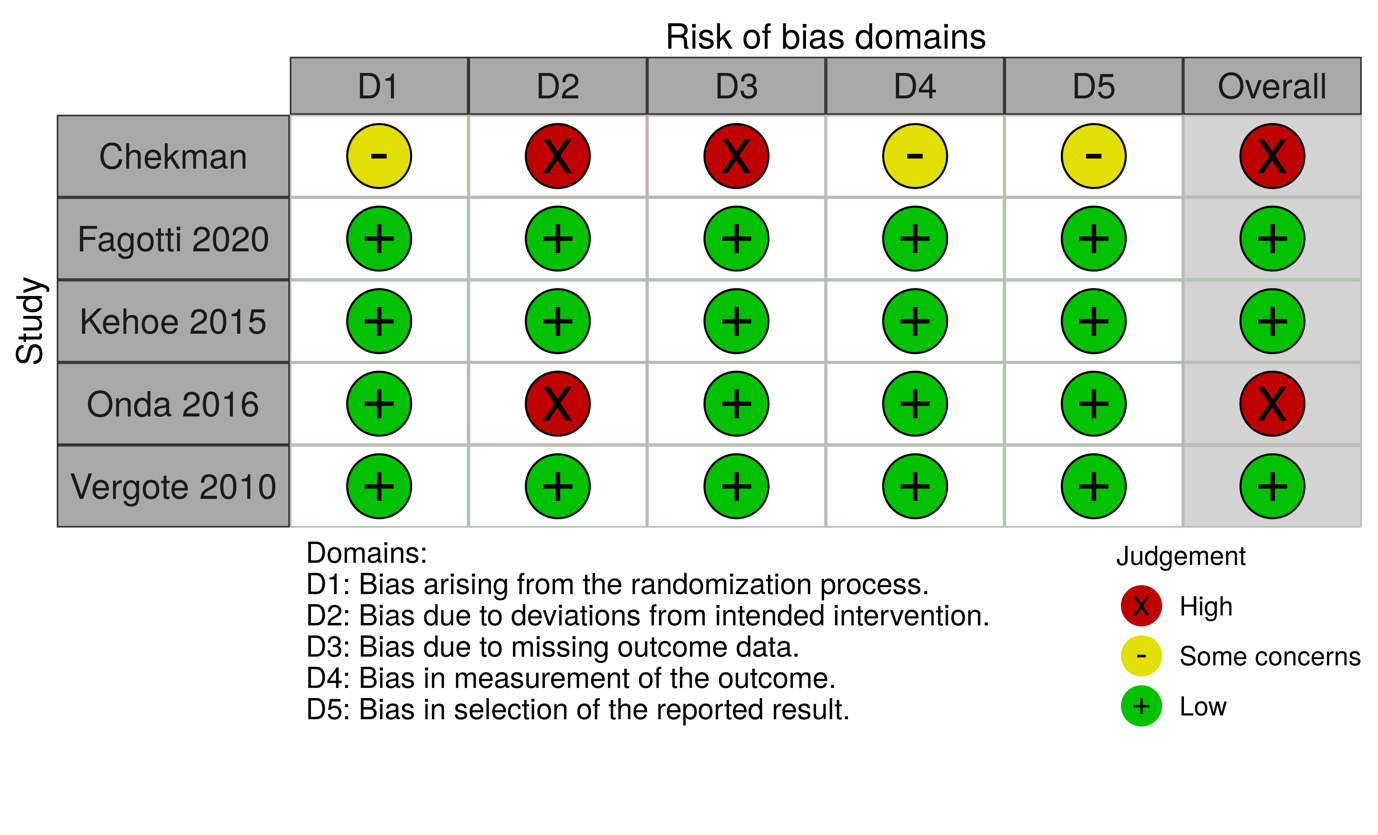


Figure 4. Peri-operative adverse events, traffic-light plot.


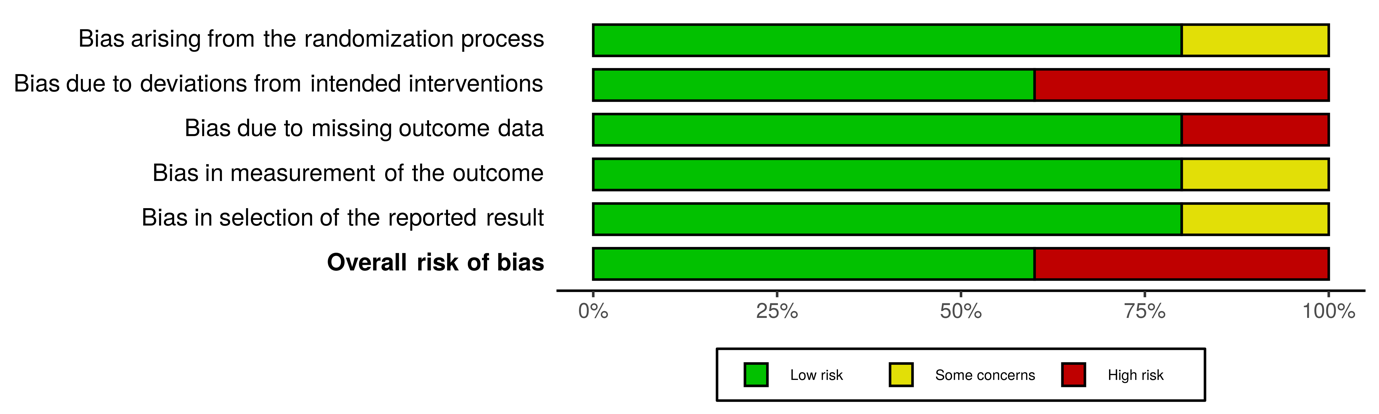


Figure 5. Peri-operative adverse events, summary plot.


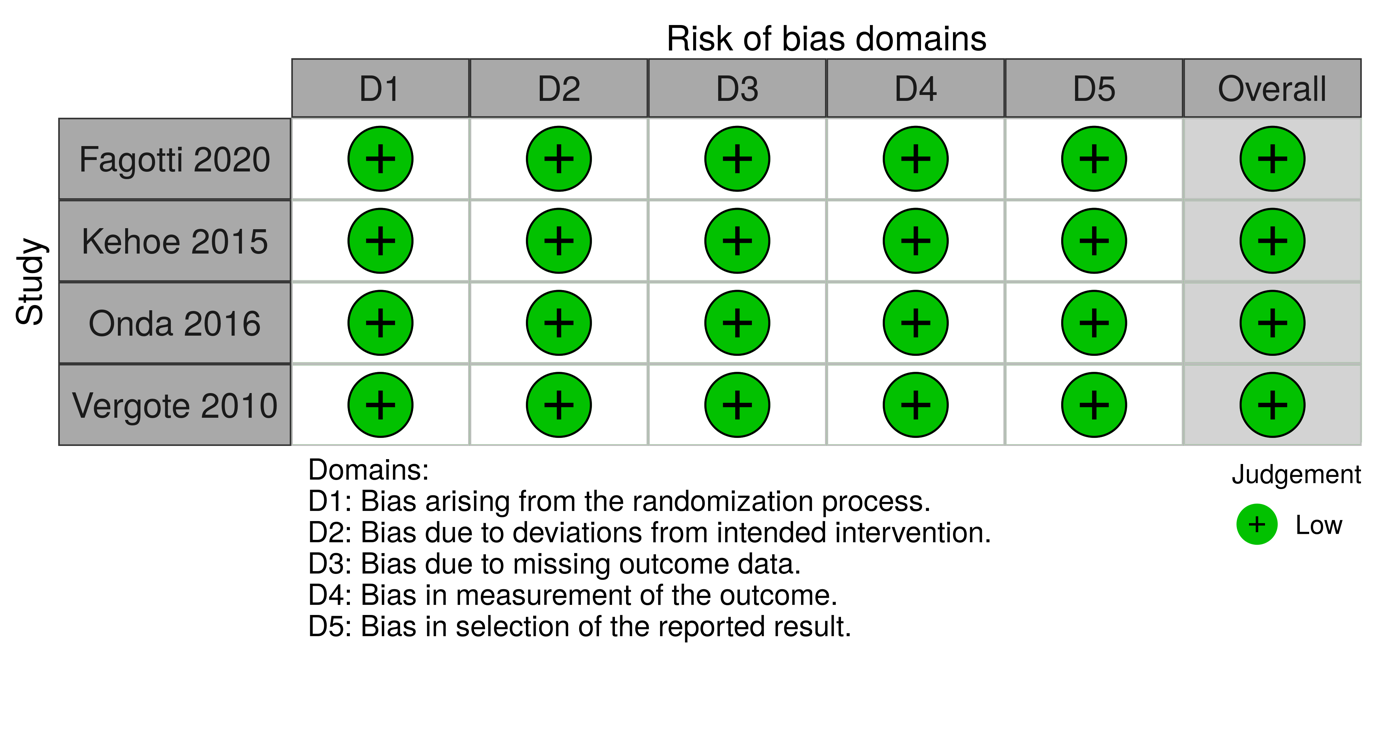


Figure 6. Cytoreduction rates, traffic-light plot.


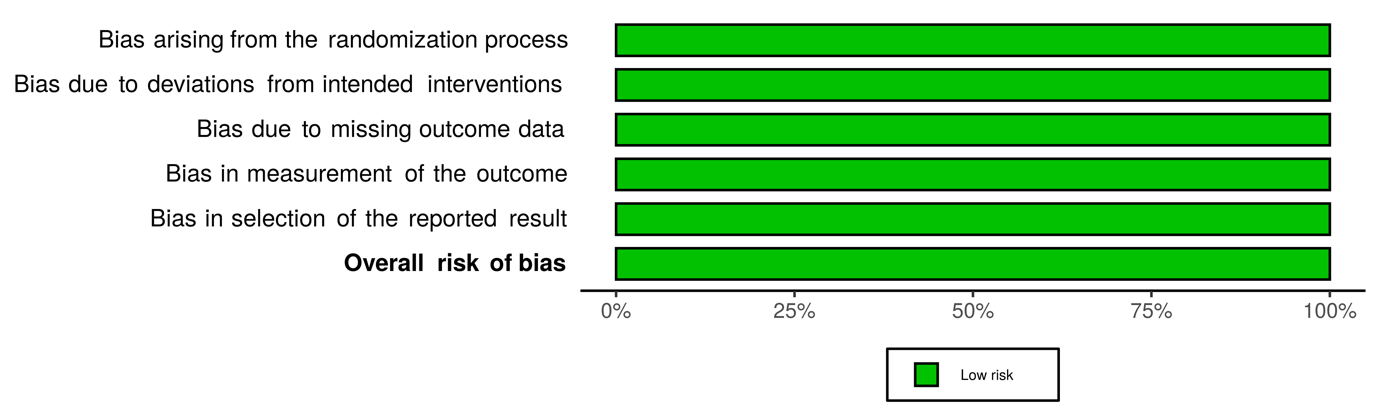


Figure 7. Cytoreduction rates, summary plot.


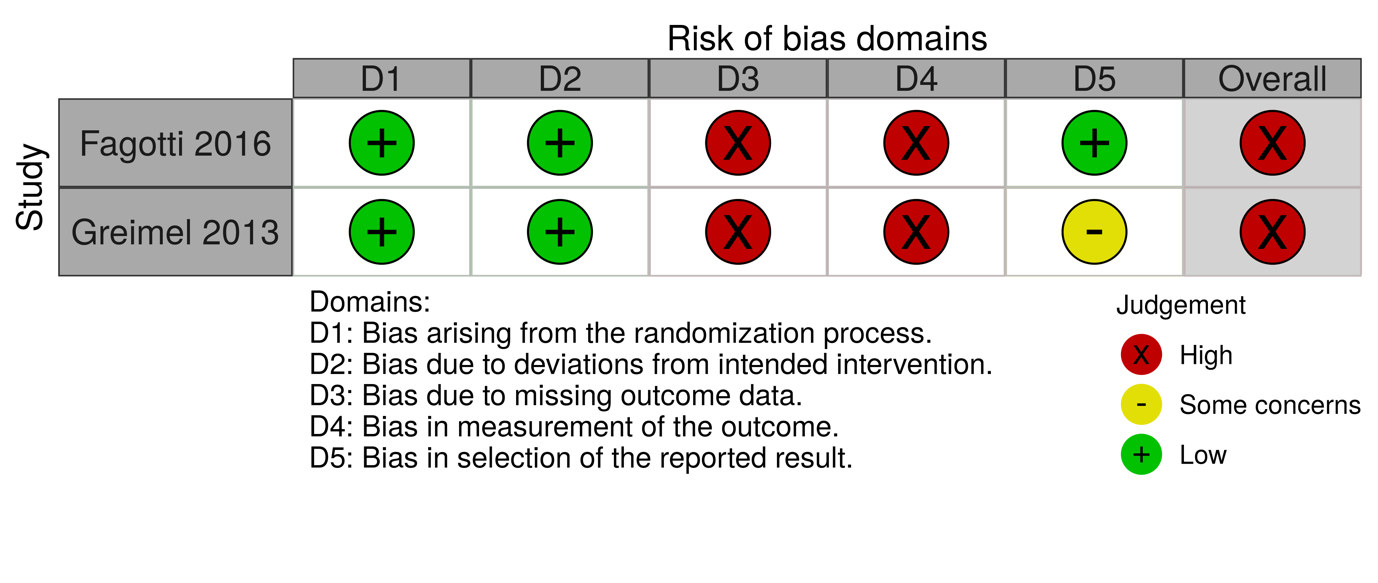


Figure 8. Quality of life, traffic-light plot.


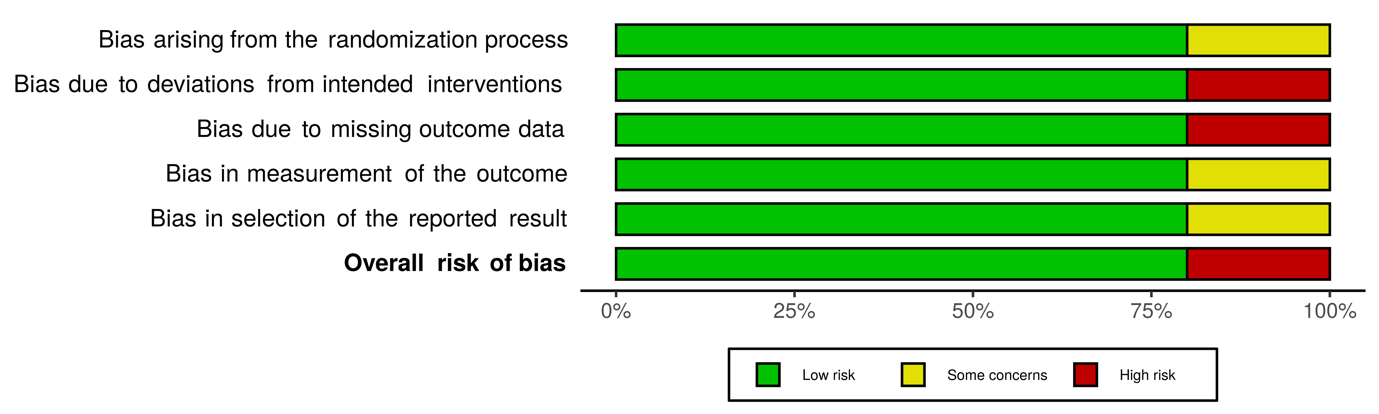


Figure 9. Quality of life, summary plot.

# Supplementary forest plots


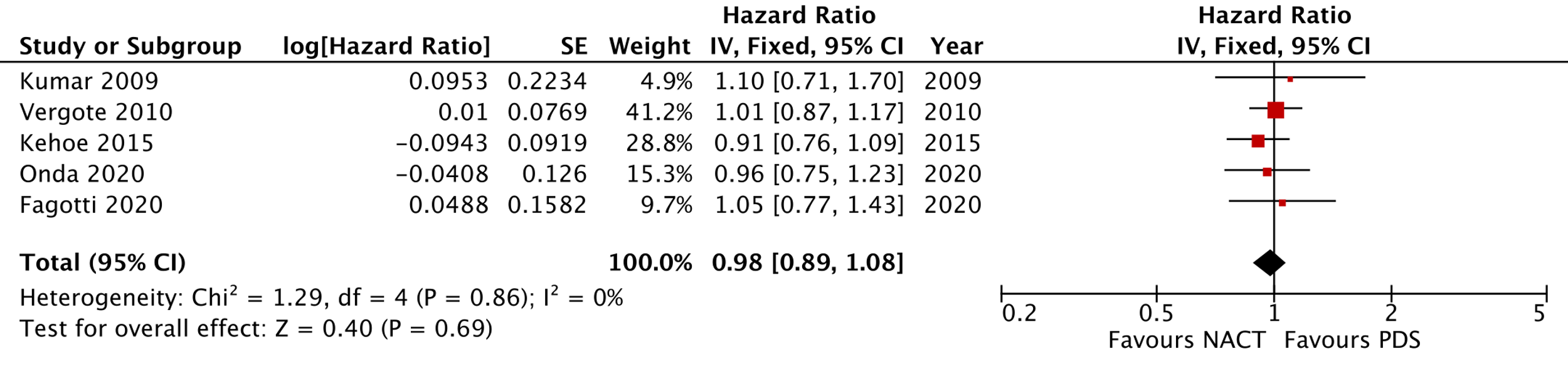


Figure 10. Progression-free survival


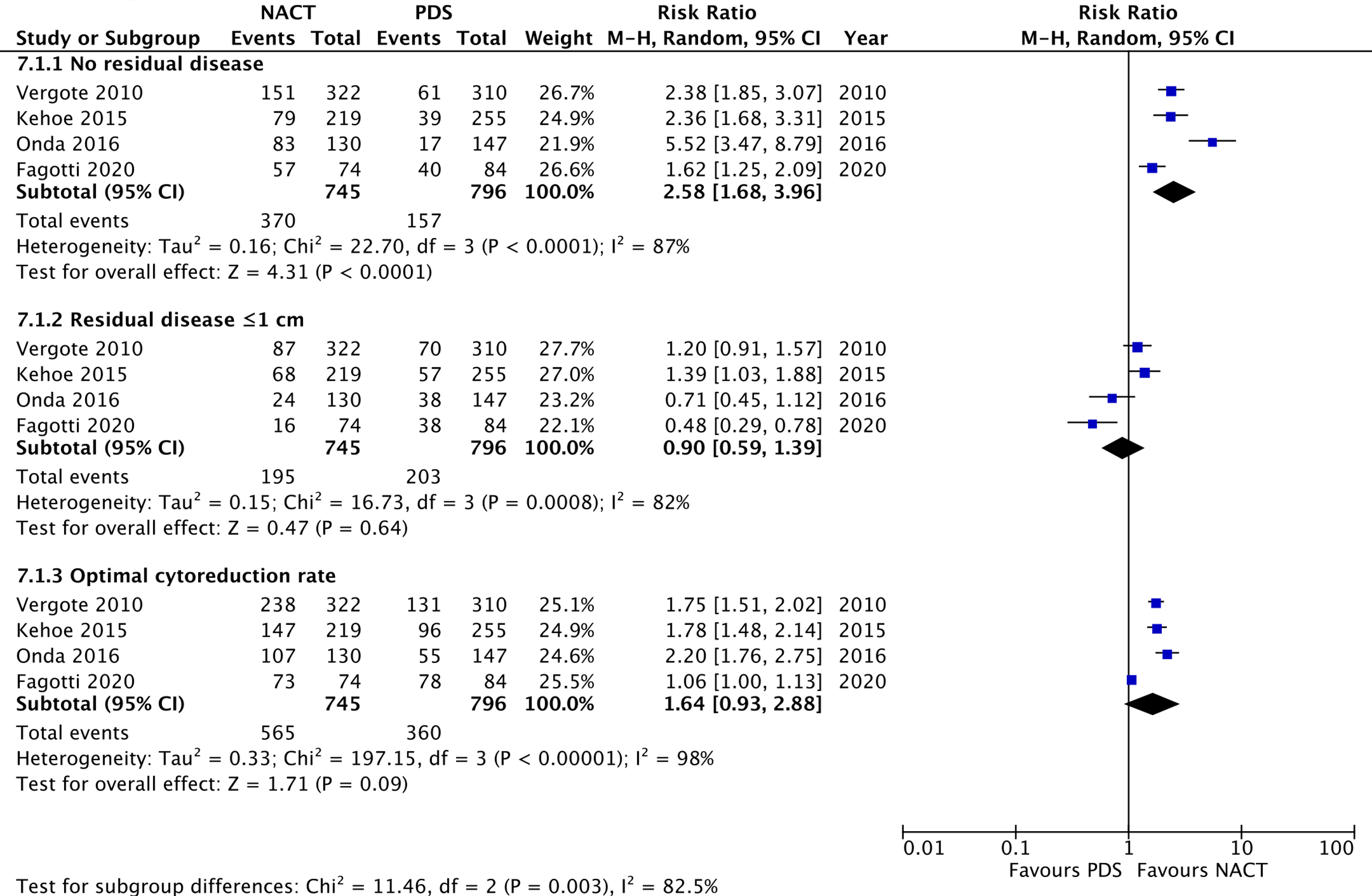


Figure 11. Extent of residual disease after cytoreductive surgery. 'As treated' analysis.


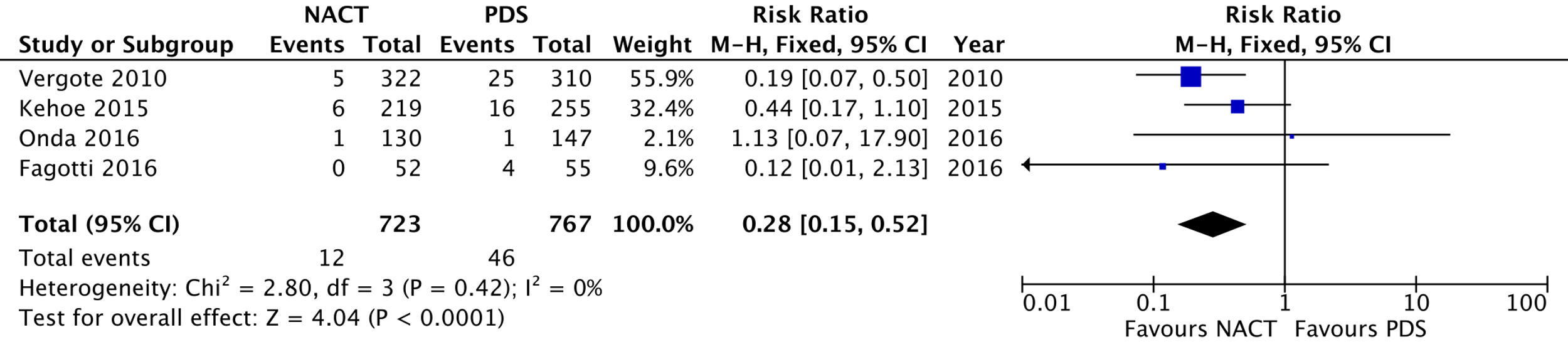


Figure 12. Infection grade 3/4.


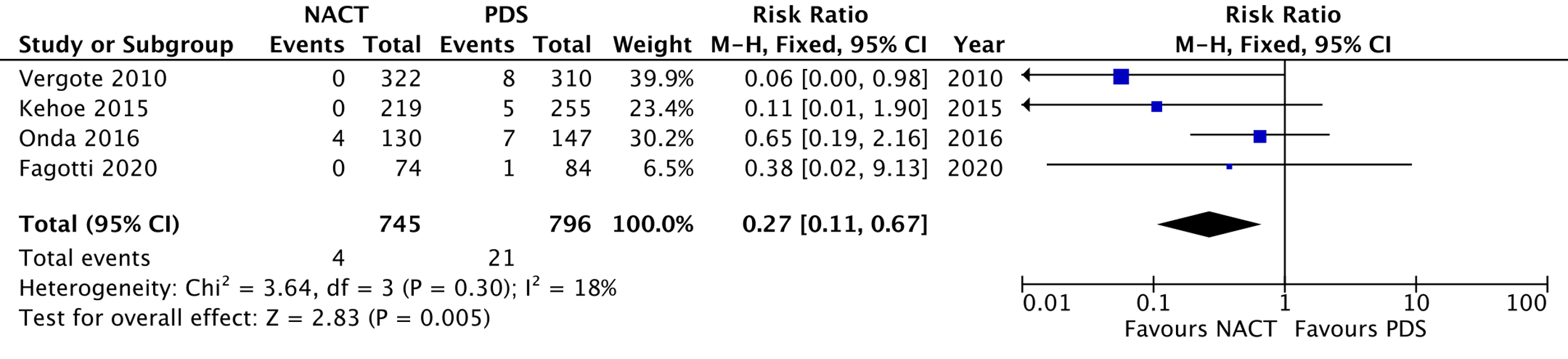


Figure 13. Venous thromboembolism.


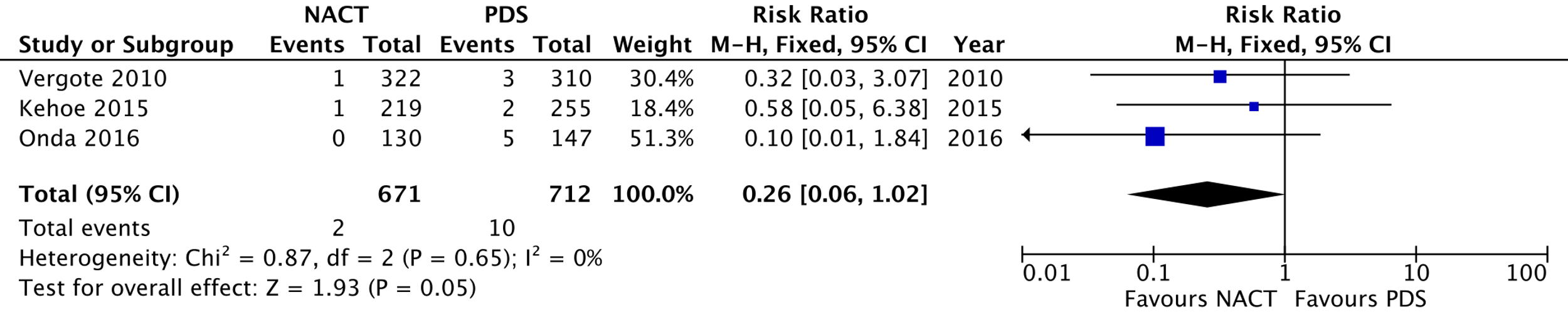


Figure 14. Gastrointestinal fistula formation.


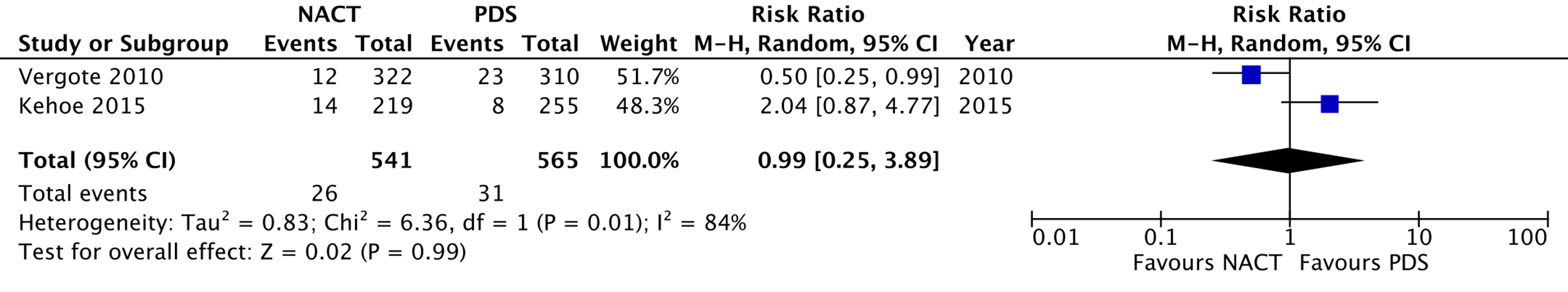


Figure 15. Haemorrhage.


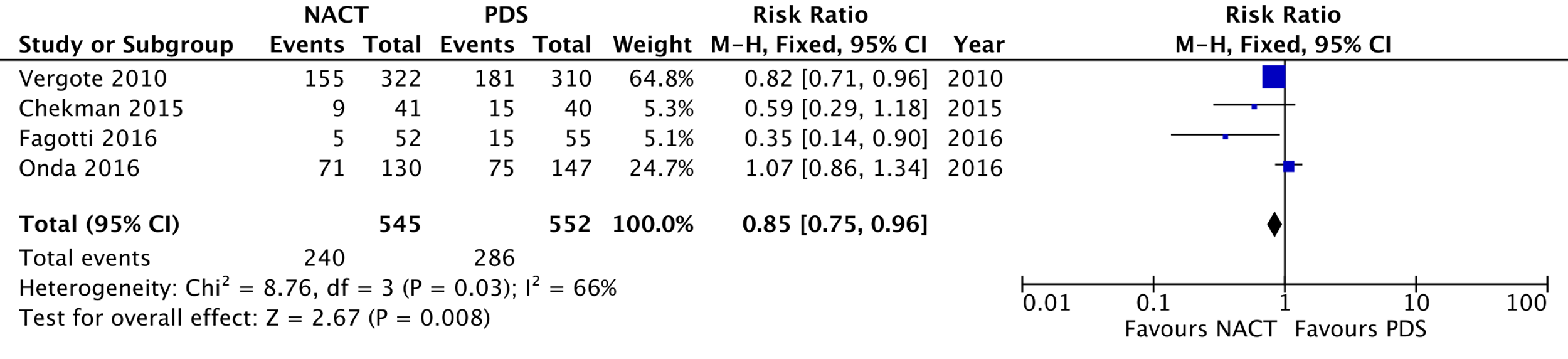


Figure 16. Blood transfusion.


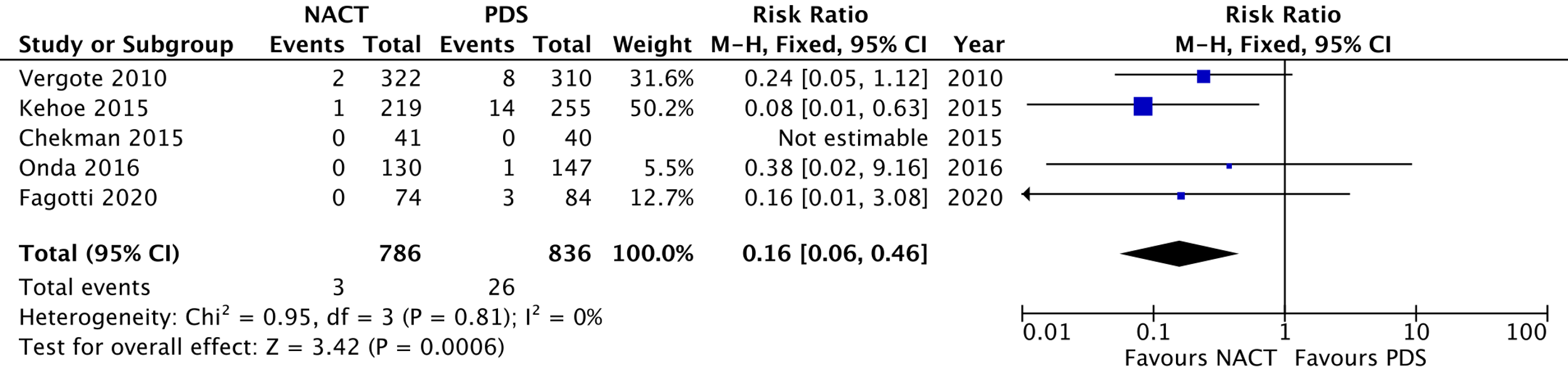


Figure 17. Post-operative mortality within 28 days.


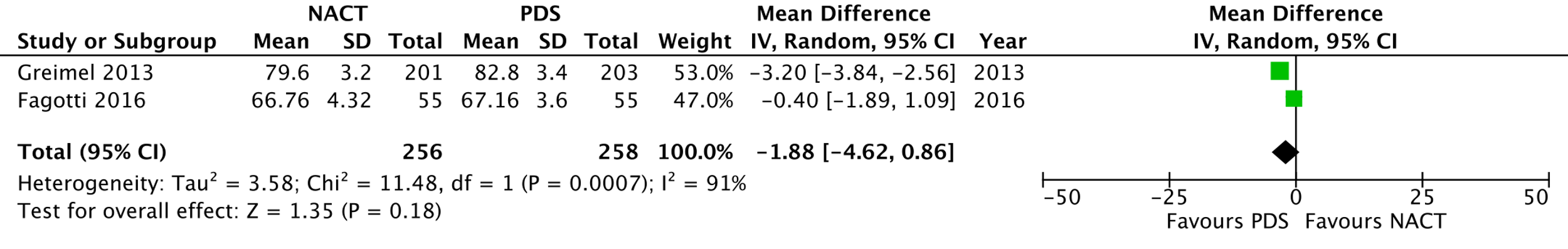

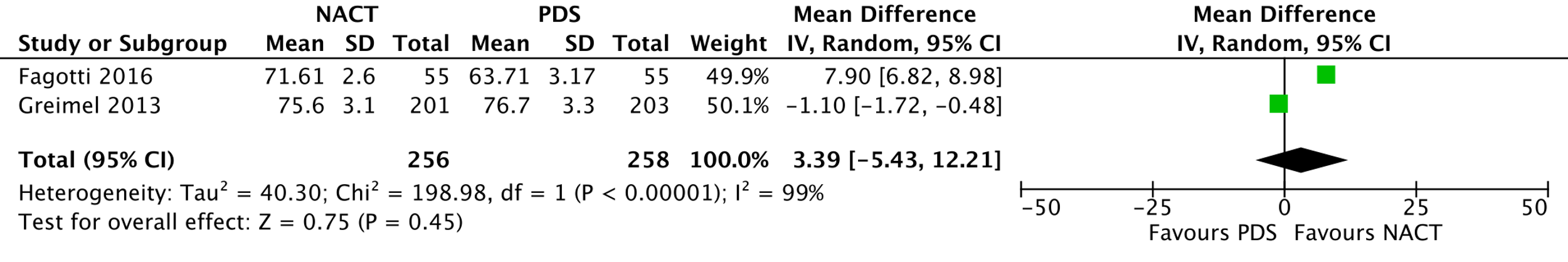


Figure 19. Physical functioning at 6-month follow-up.

Figure 18. Physical functioning at 6^th^ cycle.


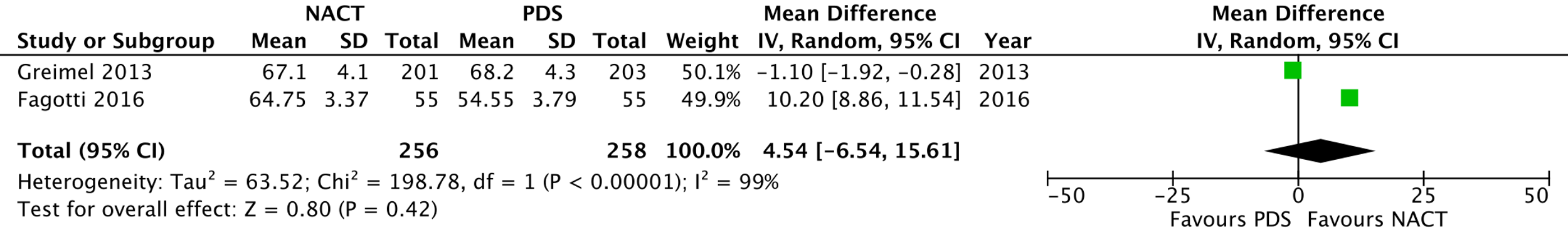


Figure 20. Role functioning at 6^th^ cycle.


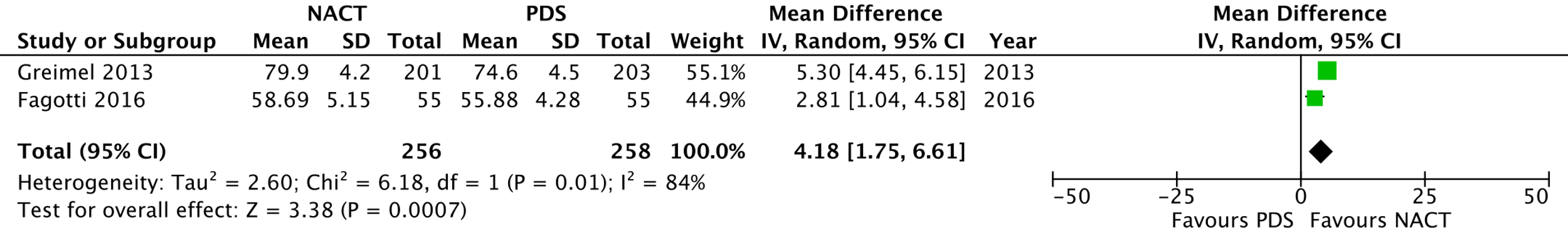


Figure 21. Role functioning at 6-month follow-up.


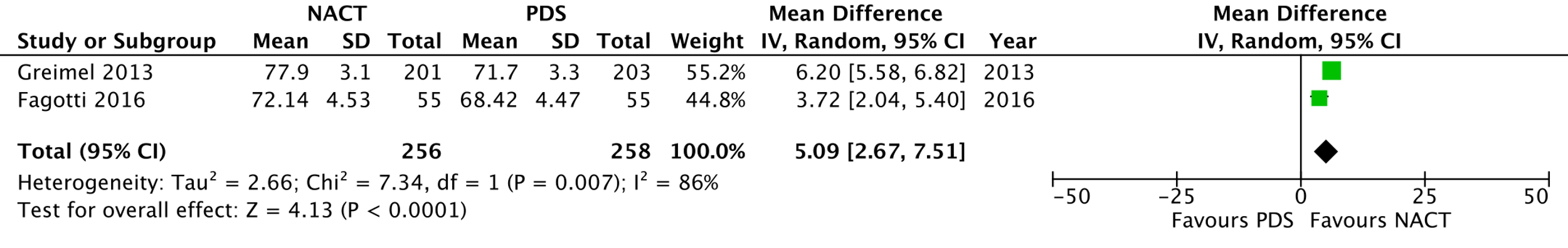

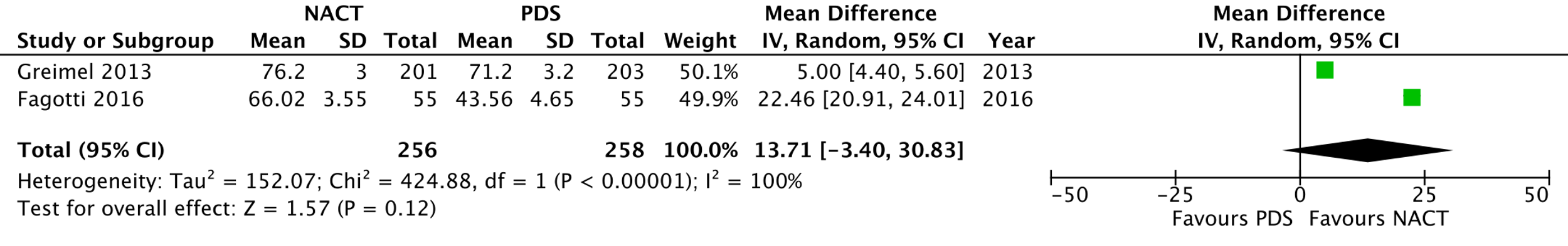


Figure 23. Emotional functioning at 6-month follow-up.

Figure 22. Emotional functioning at 6^th^ cycle.


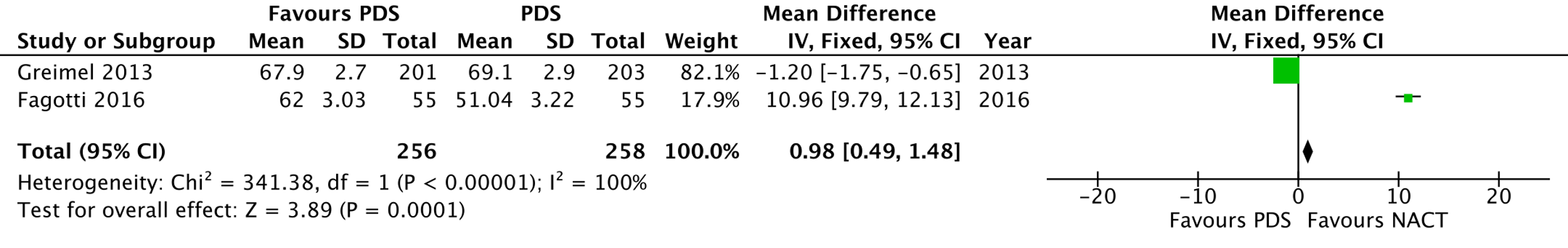


Figure 24. Global health at 6^th^ cycle.


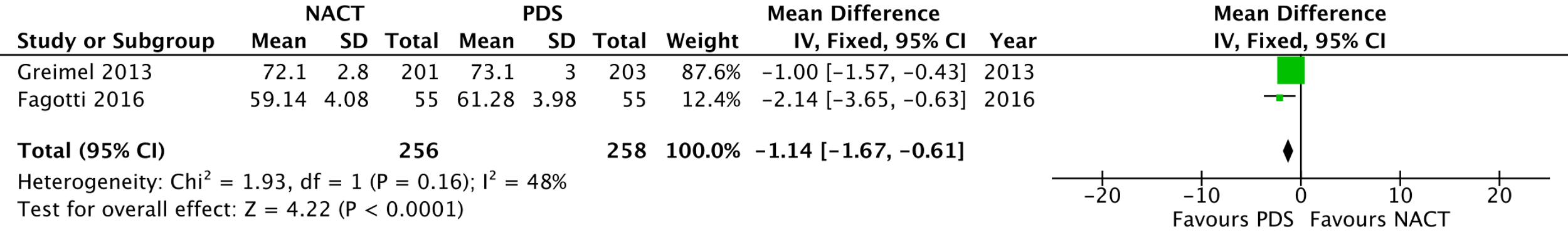


Figure 25. Global health at 6-month follow-up.


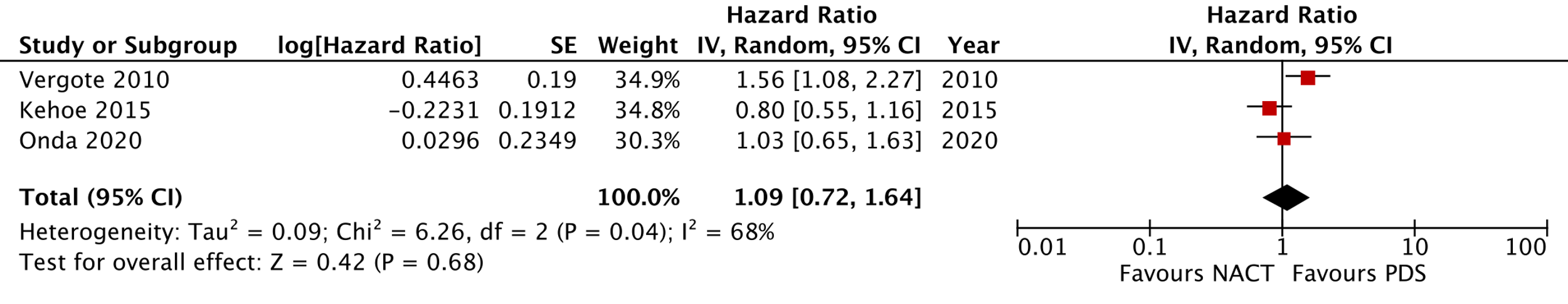

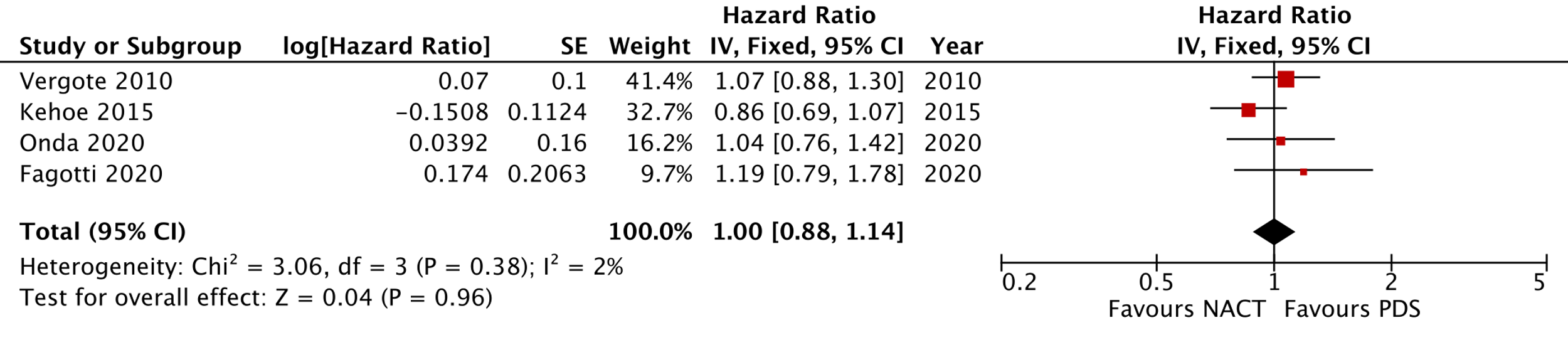


Figure 27. Overall survival in the subgroup of patients with metastatic lesions ≤5 cm in diameter,

Figure 26. Overall survival in the subgroup of patients with stage III disease.

# Trial sequential analyses

Graph 1. Trial sequential analysis. Complete cytoreduction (no residual disease).
 IDS: Interval debulking surgery performed in the neoadjuvant chemotherapy arm
 PDS: Primary debulking surgery

Graph 2. Trial sequential analysis. Any grade 3/4 peri-operative adverse event.
